# Comparing Pulmonary Telerehabilitation and Center-Based Pulmonary Rehabilitation for Effectiveness and Adherence in Chronic Obstructive Pulmonary Disease: Systematic Review and Meta-Analysis of Randomized Controlled Trials

**DOI:** 10.2196/80500

**Published:** 2026-04-17

**Authors:** Ya Li, Hailong Zhang, Guixiang Zhao, Jiansheng Li, Haoxuan Huang, Longyu Wang, Peilin Jia

**Affiliations:** 1 Lung Disease Diagnosis and Treatment Center, National Medical Center, The First Affiliated Hospital of Henan University of Chinese Medicine, 19 Renmin Road, Zhengzhou, Henan, China, 86 13643718969; 2 Collaborative Innovation Center for Chinese Medicine and Respiratory Diseases Co-constructed by Henan Province & Ministry of Education of P.R. China, Henan Key Laboratory of Chinese Medicine for Respiratory Diseases, Henan University of Chinese Medicine, Zhengzhou, Henan, China

**Keywords:** chronic obstructive pulmonary disease, Tele-PR, pulmonary rehabilitation, digital health, meta-analysis

## Abstract

**Background:**

Pulmonary rehabilitation (PR) is a cornerstone of chronic obstructive pulmonary disease (COPD) management; however, access to traditional center-based PR (CBPR) remains limited. Digital and remote models, collectively termed pulmonary telerehabilitation (Tele-PR), have increasingly been used, but their heterogeneity in technology use, supervision, and interaction mode may influence effectiveness and sustainability.

**Objective:**

This systematic review and meta-analysis aimed to compare the effectiveness and adherence of Tele-PR with those of CBPR in adults with COPD while systematically evaluating the impacts of supervision intensity and delivery models on key clinical outcomes.

**Methods:**

This review followed PRISMA (Preferred Reporting Items for Systematic Reviews and Meta-Analyses) 2020 and PRISMA-S (Preferred Reporting Items for Systematic reviews and Meta-Analyses literature search extension) guidelines. PubMed, Embase, the Cochrane Library, and the Web of Science were searched from inception to December 10, 2025, to identify randomized controlled trials comparing Tele-PR or home-based PR (HBPR) with CBPR in adults with COPD. Random effects meta-analyses were conducted using the Hartung-Knapp-Sidik-Jonkman method. Between-study heterogeneity was assessed using τ², *I²*, and 95% prediction intervals. Risk of bias was evaluated with the Cochrane Risk of Bias 2 tool, and certainty of evidence was graded using the GRADE (Grading of Recommendations Assessment, Development, and Evaluation) approach.

**Results:**

Seventeen randomized controlled trials involving 1658 participants were included. After intervention, Tele-PR and CBPR showed comparable average effects on exercise capacity by 6-minute walk distance (k=9; n=950, 57.3%; mean difference –5.37 m, 95% CI –15.68 to 4.95; *P*=.26; τ²=103.97; *I²*=28.2%; 95% prediction intervals=–32.73 to 22.27). Although pooled effects were not statistically significant, substantial heterogeneity was observed across remote delivery models. Subgroup analyses linked digitally supported, synchronously supervised Tele-PR to less between-study variance across several outcomes, indicating greater consistency in treatment effects across different settings while revealing that low-technology HBPR yielded more variable outcomes, particularly in symptom burden. At long-term follow-up (≥6 mo), between-group differences in functional and symptom outcomes diminished, and short-term gains in exercise capacity did not consistently translate into increased daily physical activity. Certainty of evidence ranged from moderate to very low, mainly downgraded for performance bias, inconsistency across intervention models, and imprecision.

**Conclusions:**

Tele-PR may achieve short-term clinical outcomes comparable to CBPR. Distinct from prior reviews, we stratified remote programs by delivery models and supervision, identifying digitally supported Tele-PR and low-technology HBPR as 2 clinically distinct paradigms with differing consistency of effects. We further propose a structured “supervision gradient” to interpret model-dependent variability in effects across Tele-PR approaches, providing a context-sensitive framework for evidence-informed, model-specific implementation. Future remote rehabilitation should integrate real-time professional supervision and long-term behavioral maintenance to sustain benefits. Tele-PR may be particularly valuable for expanding PR access, while CBPR remains essential for patients requiring close in-person supervision or complex multidisciplinary care.

## Introduction

Chronic obstructive pulmonary disease (COPD) is a progressive respiratory disorder characterized by persistent airflow limitations, commonly associated with chronic systemic inflammation, small-airway dysfunction, and emphysema [1]. Owing to its high prevalence, mortality, and disability burden, COPD imposes a substantial public health burden and is recognized as a major chronic condition threatening global health. It is projected that, by 2030, COPD will account for approximately 4.5 million deaths annually, making it the third leading cause of death worldwide. As the disease progresses, severe dyspnea and reduced exercise tolerance may emerge, significantly impairing activities of daily living [2]. Pulmonary rehabilitation (PR) is a comprehensive, individualized intervention based on a thorough patient assessment. PR has been widely incorporated into clinical management guidelines for COPD as the most effective nonpharmacological approach for alleviating dyspnea, improving health status, and enhancing exercise capacity [3,4].

PR is typically delivered by a multidisciplinary team. Current guidelines recommend that, over a 6‐ to 8-week program, patients should attend at least two individualized assessment and training sessions per week. To optimize outcomes, programs should include endurance training, resistance training, and targeted exercises for upper and lower limbs [5]. However, due to high medical costs, health system constraints, and geographic barriers, many patients fail to achieve the expected benefits of PR [6]. Globally, fewer than 2% of eligible patients are estimated to have access to PR services, highlighting the urgent need for effective interventions to improve PR uptake [7]. PR can be implemented in various settings. Pulmonary telerehabilitation (Tele-PR) delivers exercise training, education, and self-management support through synchronous videoconferencing or asynchronous platforms and has the potential to overcome geographic and transportation barriers, thereby enhancing access to rehabilitation services [8-10].

Randomized controlled trials (RCTs) have shown that, when frequency and intensity are comparable, community- and home-based rehabilitation programs achieve outcomes similar to those of hospital-based programs [11]. The current evidence base is no longer limited to COPD. Recent studies in the broader field of respiratory rehabilitation have provided strong support for the effectiveness of remote interventions. In an RCT, Sánchez-Romero et al [12] reported that an evidence-based eHealth educational tool combined with rehabilitation training yielded significant improvements in musculoskeletal symptoms and patient adherence. In addition, a network meta-analysis by Martínez-Pozas et al [13,14] confirmed that Tele-PR was noninferior to face-to-face rehabilitation in improving physical function and quality of life and produced comparable effects in reducing dyspnea and fatigue. These successful applications in complex respiratory conditions provide supportive evidence for the feasibility and potential effectiveness of implementing Tele-PR in COPD.

Nevertheless, several key uncertainties remain in the current evidence regarding Tele-PR in COPD. First, prior systematic reviews have frequently pooled remote interventions with varying levels of technology, supervision intensity, and interaction modalities, thereby implicitly assuming that Tele-PR is relatively homogeneous—a presumption that may obscure important differences across implementation models. Second, evidence on the long-term maintenance effects of Tele-PR remains limited, particularly regarding whether postintervention improvements in exercise capacity translate into sustained changes in daily physical activity, for which consistent conclusions are still lacking. Third, the potential influence of different supervision modalities on safety, consistency of treatment effects, and patient behavioral responses has not been systematically elucidated.

To address these gaps, this systematic review and meta-analysis was designed to comprehensively compare Tele-PR with center-based pulmonary rehabilitation (CBPR) in patients with COPD. The objectives were to (1) evaluate the comparative effectiveness of Tele-PR versus CBPR on exercise capacity, symptom burden, and health-related quality of life; (2) stratify remote rehabilitation by supervision intensity and technological features to compare synchronously supervised, digitally supported Tele-PR with traditional low-technology home-based PR (HBPR); and (3) examine outcome trajectories at the end of the intervention and during long-term follow-up to inform evidence-based implementation and resource allocation.

## Methods

### Registration

This systematic review and meta-analysis was conducted and reported in accordance with the PRISMA (Preferred Reporting Items for Systematic Reviews and Meta-Analyses) 2020 statement [15] and the PRISMA-S extension for literature searches. An expanded PRISMA 2020 checklist, the PRISMA 2020 checklist for abstracts, and the PRISMA-S checklist [16] are provided in Checklist 1. The structure of the Methods section follows the corresponding PRISMA 2020 headings to enhance transparency, reproducibility, and suitability for editorial review. Protocol changes are detailed in Multimedia Appendix 1.

### Search Strategy

A comprehensive literature search was performed in PubMed (National Library of Medicine), Embase (Elsevier), the Cochrane Library (Wiley), and the Web of Science (Clarivate) from database inception to October 15, 2024, with an updated search conducted on December 10, 2025. The search strategy was independently developed de novo and refined by 2 reviewers to identify RCTs comparing digitally supported Tele-PR or low-technology HBPR with CBPR in adults with COPD. Controlled vocabulary terms (eg, MeSH and Emtree) and free-text keywords were combined using Boolean operators. Searches were limited to articles published in English, but no restrictions were applied based on publication date or publication status (Multimedia Appendix 2). Although specific gray literature databases and clinical trial registries were not searched, we manually screened the reference lists of included studies and relevant systematic reviews to identify additional eligible trials. Furthermore, corresponding authors were contacted to request missing or unpublished data where necessary; however, no additional data were obtained. The reasons for exclusion at the full-text screening stage are reported in Multimedia Appendix 3.

### Eligibility Criteria

Study eligibility was defined according to the PICOS (population, intervention, comparator, outcomes, and study design) framework. Eligible studies were RCTs enrolling adult participants (aged ≥18 y) with COPD diagnosed according to the Global Initiative for Obstructive Lung Disease criteria (postbronchodilator forced expiratory volume in 1 s [FEV₁]/forced vital capacity of <0.70), regardless of Global Initiative for Obstructive Lung Disease stage (I to IV) or exacerbation history. Tele-PR interventions were eligible if they could be classified a priori into one of 2 conceptually distinct models based on technological infrastructure and supervision intensity. Digitally supported Tele-PR was defined as any comprehensive PR program delivered primarily in the home setting and supported by information and communication technology, enabling real-time or asynchronous interaction with health professionals (eg, synchronous videoconferencing, mobile apps, web-based platforms, or wearable-supported monitoring with structured feedback), while HBPR encompassed lower-technology home programs relying primarily on structured telephone coaching and/or printed training materials, provided that systematic professional guidance or supervision was delivered (ie, not fully unsupervised self-training). To harmonize heterogeneous Tele-PR implementations, we used a prespecified hierarchical taxonomy: delivery models (digitally supported Tele-PR vs low-technology HBPR), supervision modality (synchronous real-time supervised sessions vs asynchronous delayed feedback or periodic contacts), and supervision intensity (high: ≥2 structured contacts per week; medium: ~1 per week; and low or minimal: <1 per week or instruction only). These categories were defined based on trial descriptions and were used for subgroup and exploratory analyses. Meanwhile, the comparator was CBPR, which was defined as any standardized PR program delivered in hospital or community settings with direct, in-person professional supervision. Studies were required to report at least one prespecified outcome (eg, 6-min walk distance [6MWD], COPD Assessment Test [CAT], St. George’s Respiratory Questionnaire [SGRQ], Chronic Respiratory Questionnaire, modified Medical Research Council Dyspnea scale, Hospital Anxiety and Depression Scale, daily step count, self-efficacy, dropout, adverse events, or exacerbations). Outcomes reported by only a small number of studies were synthesized descriptively or exploratorily.

Studies were excluded if they (1) used nonrandomized designs (eg, observational studies); (2) were reviews, case series, editorials, protocols, or conference abstracts without accessible full texts; (3) enrolled participants with contraindications to exercise due to severe cardiovascular, musculoskeletal, or other limiting comorbidities; or (4) evaluated interventions not primarily designed for COPD rehabilitation or involved mixed programs in which the specific effects of Tele-PR or HBPR could not be isolated.

### Study Selection and Data Extraction

Study selection and data extraction were performed independently by 2 teams using a standardized workflow. Duplicates were removed in EndNote 21 (Clarivate), after which titles or abstracts and full texts were screened in duplicate. Interrater agreement was assessed using Cohen κ [17], indicating substantial agreement for title or abstract screening (κ=0.75) and almost perfect agreement for full-text eligibility assessment (κ=0.92). Disagreements were resolved through discussion with a third reviewer. Backward citation tracking of included studies was conducted as a supplementary search step after full-text screening. Key study characteristics (eg, first author, year, sample size, design, participant characteristics, intervention details, and outcomes) were extracted in duplicate; missing data were requested from corresponding authors when necessary, and discrepancies were resolved through discussion with a third researcher.

### Data Analysis

We pooled dichotomous outcomes using risk ratios and continuous outcomes using mean differences (MDs), as outcomes were measured on consistent scales. Meta-analyses were conducted in R (version 4.3.1; R Foundation for Statistical Computing) using the meta package (version 7.0‐0). Given the anticipated clinical and methodological heterogeneity across interventions, populations, and delivery models, all meta-analyses were conducted using random effects models, irrespective of the magnitude of statistical heterogeneity. This choice was conceptual rather than data-driven and reflects the assumption that true effects may vary across settings. Pooled effect estimates were calculated using the Hartung-Knapp-Sidik-Jonkman (HKSJ) method, which provides more robust CIs and reduces the risk of false-positive findings, particularly when the number of included studies is small [18]. Between-study variance (τ²) was estimated using the Sidik-Jonkman estimator. When outcomes were reported at multiple time points, separate meta-analyses were conducted for the end-of-intervention and long-term follow-up data (≥6 mo), as these time points address distinct clinical questions regarding short-term efficacy and durability of effects. For the primary meta-analyses, 95% PIs were calculated using the confidence distribution approach proposed by Nagashima et al [19] via the pimeta package. This approach accounts for uncertainty in the estimation of between-study variance and improves coverage probability compared with conventional normal-based methods [20]. While *I²* values are reported descriptively, interpretation of heterogeneity primarily emphasized PIs, given their greater relevance for real-world applicability. Meta-analyses were not conducted when fewer than 3 studies were available, as estimation of between-study heterogeneity and prediction intervals (PIs) would be statistically unreliable; such outcomes were synthesized narratively. To improve numerical stability for PI estimation, step counts were rescaled (per 100 steps per day) without changing standardized interpretation. Where appropriate (k≥10 studies), we assessed small-study effects using contour-enhanced funnel plots, Egger test (for continuous outcomes), and Peters linear regression test (for binary outcomes), interpreting these as indicators of small-study effects rather than sole proof of publication bias [21,22]. To explore potential sources of heterogeneity, meta-regressions for continuous moderators were visualized using bubble plots, whereas subgroup analyses for categorical moderators were summarized in tabular format to provide detailed group-specific estimates. These analyses are inherently observational and explore potential moderators rather than establish causality [23]. Leave-one-out sensitivity analyses were conducted for all primary outcomes to assess the robustness of pooled estimates and to identify influential studies contributing to between-study heterogeneity. Multiple reports from the same trial were treated as a single study unit, and no study contributed more than one effect size to the same meta-analysis. For multiarm trials, only one eligible comparison per study was included, preventing the duplicate use of shared control groups [24].

### Quality Assessment

Methodological quality was assessed using the Cochrane Risk of Bias 2 tool [25]. Two reviewers independently evaluated 5 domains: randomization, deviations from intended interventions, missing outcome data, outcomes measurement, and selective reporting. Each domain was rated as low risk, some concerns, or high risk. Disagreements were resolved through discussion or consultation with a third reviewer. The certainty of evidence for primary outcomes was graded using the GRADE (Grading of Recommendations, Assessment, Development, and Evaluation) approach [26], considering risk of bias, inconsistency, indirectness, imprecision, and publication bias.

### Ethical Considerations

As this study is a systematic review and meta-analysis of previously published data, it did not involve the recruitment of human participants or access to identifiable private patient information. Consequently, institutional review board or research ethics board approval was not required. Ethical approval and informed consent were obtained by the authors of the primary studies included in this review.

## Results

### Study Characteristics

**Figure 1.**
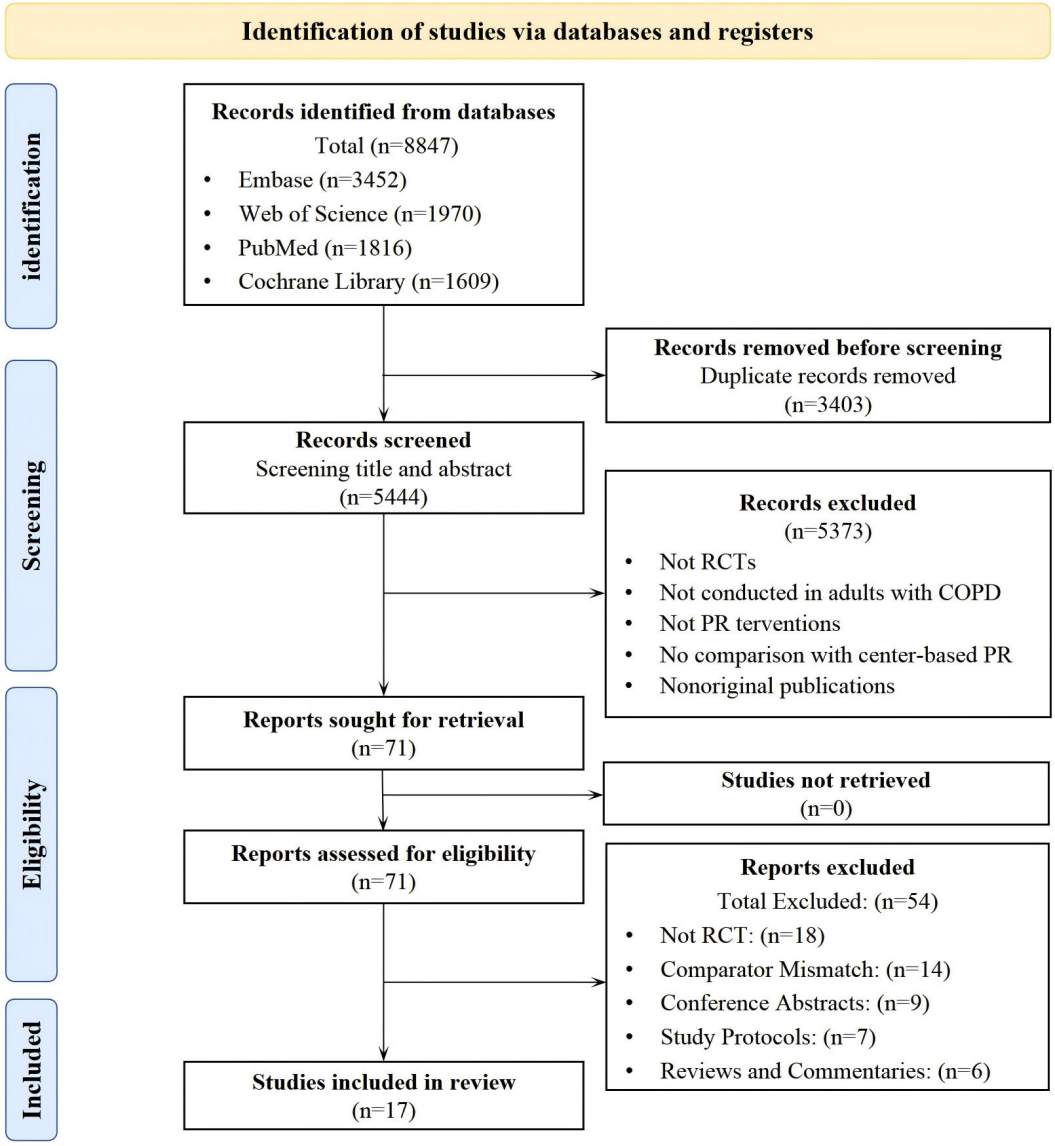
PRISMA (Preferred Reporting Items for Systematic Reviews and Meta-Analyses) flow diagram illustrating the study selection process, identifying 17 randomized controlled trials (RCTs) comparing pulmonary telerehabilitation with center-based pulmonary rehabilitation (PR) in patients with chronic obstructive pulmonary disease (COPD).

A total of 8847 records were retrieved in the initial search. After stepwise screening, 17 studies were included. The selection process is presented in a PRISMA flow diagram (Figure 1).

A total of 17 trials [27-43] were identified and included in the quantitative synthesis. To ensure statistical independence, multiple reports originating from the same trial were linked via trial registration numbers, and data were extracted from a single source per outcome to avoid participant double-counting (Multimedia Appendix 4 for a detailed audit of study independence). The included trials were published between 2008 [36] and 2025 [43]. The trials were conducted across multiple regions, including Europe (the United Kingdom [27,34,35,39], Spain [29,37,43], Denmark [30,31], and Greece [40]), Australia [28,33,38,41], Asia (China [32]), North America (Canada [36]), and South America (Brazil [42]). Sample sizes ranged from 54 participants [29] to more than 150 participants [39]. All participants were adults with a confirmed diagnosis of COPD. One trial [33] enrolled patients with chronic respiratory diseases; however, in this meta-analysis, only data from its COPD subgroup were extracted. The most studies included a higher proportion of male participants. Regarding disease severity, participants predominantly had moderate-to-severe airflow limitations. Tele-PR interventions were classified into 3 distinct models: synchronous video supervision, in which direct remote supervision was provided by physiotherapists via real-time videoconferencing [30,31,33]; asynchronous digital platform support, in which structured rehabilitation content was delivered through web-based platforms [27,34,40], mobile apps, or wearable sensors [29,32], with supervision primarily provided as data-driven asynchronous feedback; and low-technology and telephone support [28,35,36,38,39,42,43], which relied primarily on printed materials for self-management guidance and was supplemented by telephone follow-up to enhance adherence and motivation. A minimal supervised model was also reported [37]. All trials compared Tele-PR with CBPR. The intervention duration was typically 8 to 10 weeks [29,31,33,36,38,41], with some studies lasting 7 weeks [27,28,39] or extending to 12 to 13 weeks [42,43]. Several trials reported long-term follow-up data at 6 to 12 months after the intervention [30,32,33,40,41,43]. Detailed characteristics of the included RCTs are summarized in Table 1. Intervention components and supervision models are detailed in Multimedia Appendix 5.

**Table 1.** Characteristics of the included randomized controlled trials.

Study	Country	Sample size (Tele-PR^a^/control)	Age (years), mean (SD)	Male, % (n/N)	FEV_1_^b^ (% predicted), mean (SD)	Tele-PR intervention (mode)	Supervision (type and frequency)	Control group (setting)	Duration
Chaplin et al (2017) [27]	UK	51/52	I^c^: 66.4 (10.1) C^d^: 66.1 (8.1)	I: 74.5%(38/51) C: 63.5%(33/52)	I: 59 (29) C: 55 (21)	Web based (interactive website)	Asynchronous: weekly emails or calls for remote support	Hospital or community PR^e^	7 weeks
Holland et al (2017) [41]	Australia	80/86	I: 69 (13) C: 69 (10)	I: 60.0% (48/80) C: 59.3% (51/86)	I: 52 (19) C: 49 (19)	Telephone based (health coaching)	Minimal: 1 home visit+7 weekly telephone calls	Hospital outpatient PR	8 weeks
Horton et al (2018) [39]	UK	145/142	I: 68 (9) C: 67 (8)	I: 64.1% (93/145) C: 66.2% (94/142)	I: 48 (19) C: 49 (17)	Manual based (“SPACE for COPD”)	Minimal: self-managed with manual+2 calls (weeks 2 and 4)	Hospital outpatient PR	7 weeks
Hansen et al (2020) [31]	Denmark	67/67	I: 68.4 (8.7) C: 68.2 (9.4)	I: 47.8% (32/67) C: 41.8% (28/67)	I: 33 (10) C: 34 (8)	Video based (real-time videoconferencing)	Synchronous: real-time supervision by physio via tablet	Hospital outpatient PR	10 weeks
Maltais et al (2008) [36]	Canada	126/126	I: 66 (9) C: 66 (9)	I: 54.0%(68/126) C: 57.1%(72/126)	I: 46 (13) C: 43 (13)	Home based (exercise equipment provided)	Remote: initial home visit+weekly telephone calls	Hospital outpatient PR	8 weeks
Vasilopoulou et al (2017) [40]	Greece	50/50	I: 66.9 (9.6) C: 66.7 (7.3)	I: 93.6%(44/47) C: 76.0%(38/50)	I: 50 (22) C: 52 (17)	Tablet/web (platform monitoring)	Asynchronous: data upload 3-4 times per week+ weekly feedback calls	Hospital outpatient PR	12 months
Sacristán-Galisteo et al (2025) [43]	Spain	40/40	I: 67.9 (8.8) C: 68.9 (8.2)	I: 72.5% (29/40) C: 72.5% (29/40)	I: 53 (16) C: 55 (16)	Telephone based (home exercise diary)	Remote: weekly telephone calls (15-20 min) for motivation	Health center (face-to-face)	13 weeks
Li et al (2022) [32]	China	50/49	I: 65.9 (8.9) C: 65.6 (8.8)	I: 82.0% (41/50) C: 76.0% (38/50)	I: 49 (11) C: 50 (11)	App/WeChat (smartphone uploads)	Remote: daily interaction via WeChat group (uploads and feedback)	Hospital outpatient PR	12 months
Cox et al (2022) [33]	Australia	47/50	I: 68 (9) C: 67 (9)	I: 42.3% (30/71) C: 50.7% (36/71)	I: 59 (25) C: 63 (26)	Video based (virtual group video)	Synchronous: real-time group videoconferencing via tablet	Hospital outpatient PR	8 weeks
Cerdán-de-las-Heras et al (2021) [29]	Spain	27/27	I: 67.4 (10) C: 72.5 (7)	I: 59.3% (16/27) C: 55.6% (15/27)	I: 36 (14) C: 33 (9)	App/sensor (“VAPA” app+chest sensor)	Hybrid: autonomous AI supervision+remote therapist checks	Hospital standard PR	8 weeks
Burge et al (2021) [28]	Australia	70/71	69 (10)^f^	41.8% (59/141)^g^	50 (37‐63)^h^	Telephone based (home visit+calls)	Minimal: 1 home visit+7 weekly telephone calls	Hospital outpatient PR	7 weeks
Chaplin et al (2022) [34]	UK	51/52	I: 68.3 (6.5) C: 67.4 (8.6)	I: 90.0% (18/20) C: 55.9% (19/34)	I: 54 (27) C: 56 (19)	Web based (online progress monitoring)	Asynchronous: online tracking+weekly remote contact	Hospital or community PR	8 weeks
Hansen et al (2023) [30]	Denmark	67/67	68 (9)^f^	44.8% (60/134)^g^	33.1 (9.4)	Home based (long-term follow-up)	Supervised: (see [31])	Hospital outpatient PR	62 weeks
Güell et al (2008) [37]	Spain	28/29	I: 66 (6) C: 63 (7)	100.0% (57/57)^g^	I: 39 (8) C: 38 (7)	Home based (unsupervised after instruction)	Minimal: instructions at start, then self-managed	Hospital PR	9 weeks
Lahham et al (2019) [38]	Australia	20/25	I: 67 (7) C: 68 (9)	I: 45.0% (9/20) C: 56.0% (14/25)	I: 52 (19) C: 54 (19)	Telephone based (home visit+calls)	Indirect: initial supervision then weekly calls	Supervised group training	8 weeks
Mendes de Oliveira et al (2010) [42]	Brazil	42/46	I: 66.4 (10) C: 71.3 (7)	I: 81.8% (27/33) C: 82.6% (19/23)	I: 48 (23) C: 52 (24)	Home based (unsupervised after training)	Minimal: initial training at clinic, then self-monitored	Clinic-based PR	12 weeks
Horton et al (2021) [35]	UK	26/25	I: 67 (9) C: 67 (7)	I: 68.3% (43/63) C: 67.3% (37/55)	I: 47 (18) C: 51 (19)	Manual based (similar to [39])	Minimal: same as [39] (manual+2 calls)	Center-based PR	7 weeks

a Tele-PR: pulmonary telerehabilitation.

b FEV_1_: forced expiratory volume in 1 second.

c I: intervention group (pulmonary telerehabilitation).

d C: control group (center-based PR).

e PR: pulmonary rehabilitation

f Overall.

g All.

h Median (IQR).

### Methodological Quality of Included Studies

**Figure 2.**
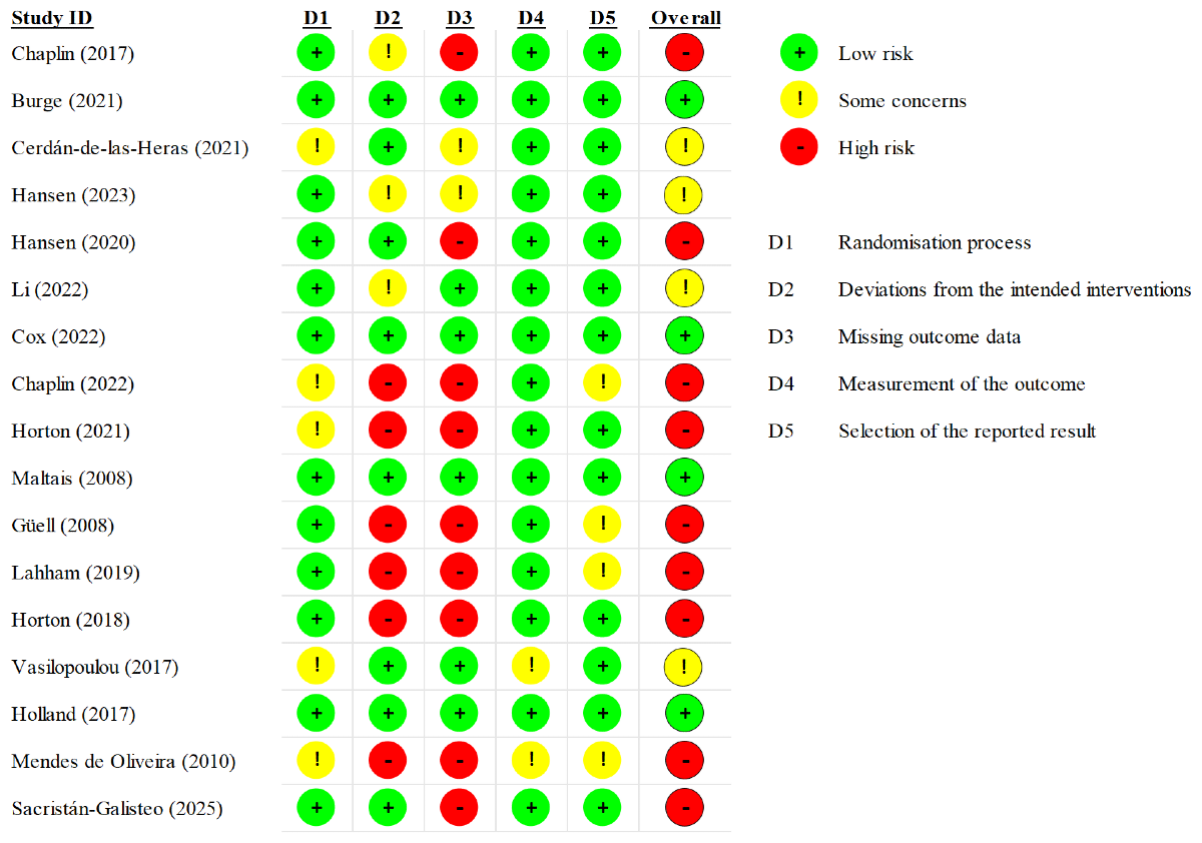
Risk of bias summary of randomized controlled trials [27-43].

The methodological quality of the included RCTs varied considerably (Figure 2). A total of 4 RCTs were rated as low risk of bias [28,33,36,41], while the remaining studies were classified as high risk or as having some concerns, mainly due to the analysis strategy they used (domain 2) or missing outcomes data (domain 3). For domain 1 (randomization process), risk was generally low, as most studies reported adequate sequence generation and allocation concealment; however, concerns were raised in a study by Cerdán-de-las-Heras et al [29] due to substantial baseline imbalance in a small sample. For domain 2 (deviations from intended interventions), risk depended on the type of analysis because blinding was difficult to implement. Studies using a strict intention-to-treat analysis were rated low risk [28,29,31,33,36,40,41,43], whereas studies relying on per-protocol analyses or completer-only analyses were judged to exhibit high risk [34,35,37-39,42]. Domain 3 (missing outcomes data) was the primary source of bias. High-risk ratings resulted from excessive dropout rates [27,39] or differential dropout between groups [31,37]. Long-term data in a study by Hansen et al [31] and Sacristán-Galisteo et al [43] were affected by substantial missingness, whereas only Cox et al [33] and Vasilopoulou et al [40] showed minimal missing data. For domain 4 (measurement of the outcome), risk was predominantly low, as blinded assessors were used for objective outcomes in most studies; Li et al [32] was an exception, as the absence of assessor blinding may have introduced detection bias. For domain 5 (selection of the reported result), risk was very low, and, in nearly all studies, reported outcomes were consistent with preregistered protocols or trial registry records. The risk of bias assessment of the included RCTs is provided in Multimedia Appendix 6.

### Meta-Analysis Results

#### Six-Minute Walk Distance

**Figure 3.**
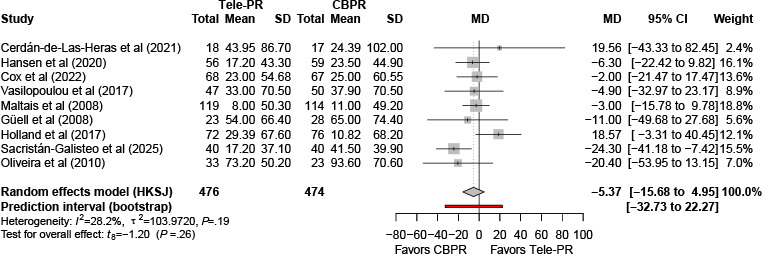
Forest plot of randomized controlled trials evaluating the effect of interventions on 6-minute walk distance in patients with chronic obstructive pulmonary disease at the end of the intervention [29,31,33,36,37,40-43]. The analysis was performed using a random effects model. CBPR: center-based pulmonary rehabilitation; HKSJ: Hartung-Knapp-Sidik-Jonkman; MD: mean difference; Tele-PR: pulmonary telerehabilitation.

Nine trials [29,31,33,36,37,40-43] provided analyzable end-of-intervention data for 6MWD. In the primary random effects meta-analysis with HKSJ adjustment, no statistically significant difference in functional capacity was observed between Tele-PR and CBPR (k=9; n=950, 57.3%; mean difference [MD] –5.37 m, 95% CI –15.68 to 4.95; *P=*.26; τ²=103.97; *I²*=28.2%; PI=–32.73 to 22.27; Figure 3).

**Figure 4.**
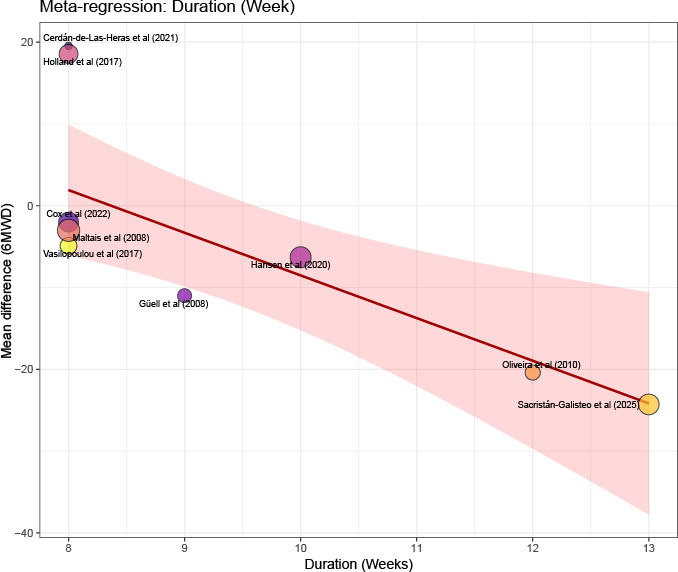
Meta-regression bubble plot of the association between intervention duration (weeks) and the mean difference in 6-min walk distance (6MWD) [29,31,33,36,37,40-43]. The size of each bubble is proportional to the weight of the study in the random effects model. The solid line represents the predicted regression line, and the shaded area indicates the 95% CI.

Potential sources of heterogeneity were explored using subgroup analyses and meta-regression. Meta-regression identified intervention duration as a significant source of heterogeneity (*β*=−5.22; *P*=.01; Table 2 and Figure 4). No significant associations were observed for economic status, publication year, training intensity, or baseline FEV_1_ (all *P>*.05; Table 2 and Multimedia Appendix 7).

**Table 2.** Meta-regression results of examining covariates influencing short-term 6-min walk distance.

Covariate	β coefficient (SE)	*t* (df)	*P* value
Economic status (developing vs developed)	−16.16 (17.78)	−0.91 (7)	.39
Intervention duration (per week)	−5.22 (1.42)	−3.68 (7)	.01
Publication year	−0.33 (0.78)	−0.42 (7)	.69
Training intensity (medium vs high)	−17.94 (10.59)	−1.69 (7)	.13
Baseline FEV_1_^a^ (% predicted)	−0.01 (0.58)	−0.02 (7)	.98

a FEV_1_: forced expiratory volume in 1 second.

**Figure 5.**
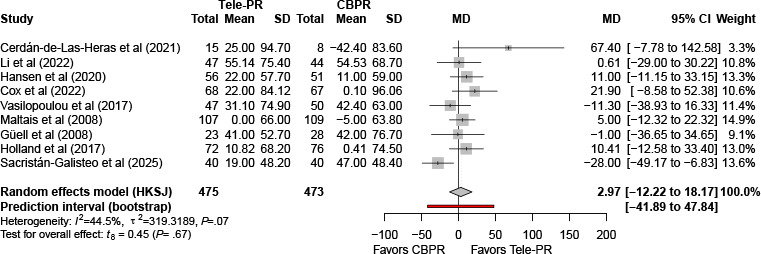
Forest plot of randomized controlled trials assessing 6-minute walk distance in patients with chronic obstructive pulmonary disease. The analysis includes outcomes at long-term follow-up (≥6 mo) [29,31-33,36,37,40,41,43]. CBPR: center-based pulmonary rehabilitation; HKSJ: Hartung-Knapp-Sidik-Jonkman; MD: mean difference; Tele-PR: pulmonary telerehabilitation.

At long-term follow-up, pooled analysis of 9 studies [29,31-33,36,37,40,41,43] also showed no statistically significant difference in 6MWD between groups (k=9; n=948, 57.2%; MD 2.97 m, 95% CI –12.22 to 18.17; *P=*.66; τ²=319.32; *I²=*44.5%; PI=−41.89 to 47.84; Figure 5).

Meta-regression analyses at long-term follow-up included 5 covariates: economic status, intervention duration, training intensity, publication year, and baseline FEV_1_. None of the covariates reached statistical significance at the .05 level (all *P>*.05; Table 3 and Multimedia Appendix 7).

**Table 3.** Meta-regression results of examining covariates influencing long-term 6-min walk distance.

Covariate	β coefficient (SE)	*t* (df)	*P* value
Economic status (developing vs developed)	−2.84 (22.81)	−0.12 (7)	.90
Publication year	−0.34 (1.20)	−0.28 (7)	.79
Intervention duration (weeks)	−6.47 (3.15)	−2.06 (7)	.08
Training intensity (medium vs high)	−3.19 (17.10)	−0.19 (7)	.86
Baseline FEV_1_^a^ (% predicted)	−0.61 (0.87)	−0.70 (7)	.51

a FEV_1_: forced expiratory volume in 1 second.

#### Daily Steps

**Figure 6.**
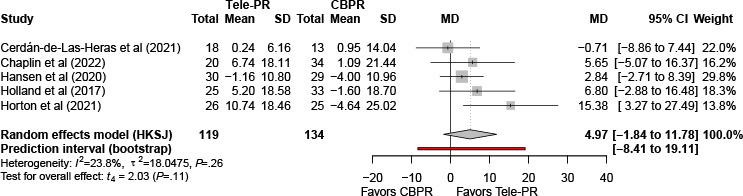
Forest plot of randomized controlled trials assessing daily step counts in patients with chronic obstructive pulmonary disease at the end of the intervention [29,31,34,35,41]. CBPR: center-based pulmonary rehabilitation; HKSJ: Hartung-Knapp-Sidik-Jonkman; MD: mean difference; Tele-PR: pulmonary telerehabilitation.

Five trials [29,31,34,35,41] reported daily step counts at the end of the intervention. The pooled analysis showed no statistically significant difference between Tele-PR and CBPR (k=5; n=253, 15.3%; MD 4.97 per 100 steps per day, 95% CI –1.84 to 11.78; *P=*.11; τ²=18.05; *I²*=23.8%; PI=–8.19 to 18.76; Figure 6).

**Figure 7.**
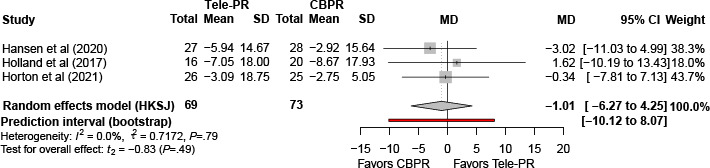
Forest plot of randomized controlled trials evaluating the effect of interventions on daily step counts in patients with chronic obstructive pulmonary disease at long-term follow-up (≥6 mo) [31,35,41]. The analysis was performed using a random effects model. CBPR: center-based pulmonary rehabilitation; HKSJ: Hartung-Knapp-Sidik-Jonkman; MD: mean difference; Tele-PR: pulmonary telerehabilitation.

At long-term follow-up, pooled analysis of 3 studies [31,35,41] showed no statistically significant overall difference (Figure 7).

#### St. George’s Respiratory Questionnaire

**Figure 8.**
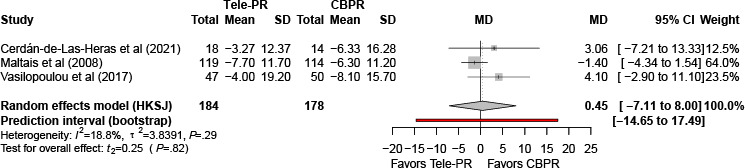
Forest plot of randomized controlled trials evaluating the effect of interventions on St. George’s Respiratory Questionnaire in patients with chronic obstructive pulmonary disease at the end of the intervention [29,36,40]. The analysis was performed using a random effects model. CBPR: center-based pulmonary rehabilitation; HKSJ: Hartung-Knapp-Sidik-Jonkman; MD: mean difference; Tele-PR: pulmonary telerehabilitation.

Three trials [29,36,40] reported SGRQ outcomes at the end of the intervention. Pooled analysis showed no statistically significant difference between Tele-PR and CBPR (k=3; n=362, 21.8%; MD 0.45, 95% CI –7.11 to 8.00; *P=*.82; τ²=3.84; *I²*=18.8%; PI=–14.65 to 17.49; Figure 8).

**Figure 9.**
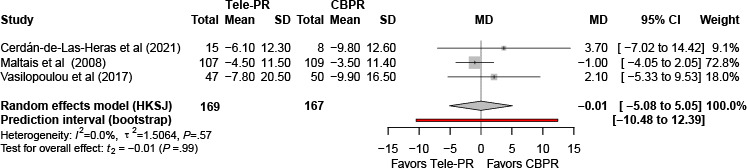
Forest plot of randomized controlled trials evaluating the effect of interventions on St. George’s Respiratory Questionnaire in patients with chronic obstructive pulmonary disease at long-term follow-up (≥6 mo) [29,36,40]. The analysis was performed using a random effects model. CBPR: center-based pulmonary rehabilitation; HKSJ: Hartung-Knapp-Sidik-Jonkman; MD: mean difference; Tele-PR: pulmonary telerehabilitation.

At long-term follow-up [29,36,40], pooled analysis likewise showed no statistically significant difference between groups (k=3; n=336, 20.3%; MD –0.01, 95% CI –5.08 to 5.05; *P>*.99; τ²=1.51; *I²*=0%; PI=–10.48 to 12.39; Figure 9).

#### COPD Assessment Test

**Figure 10.**
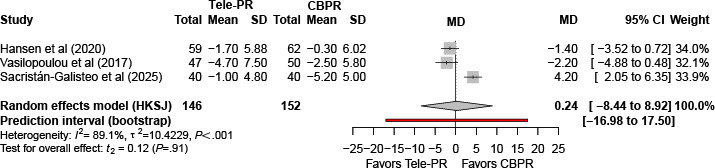
Forest plot of randomized controlled trials evaluating the effect of interventions on chronic obstructive pulmonary disease (COPD) assessment test in patients with COPD at the end of the intervention [31,40,43]. The analysis was performed using a random effects model. CBPR: center-based pulmonary rehabilitation; HKSJ: Hartung-Knapp-Sidik-Jonkman; MD: mean difference; Tele-PR: pulmonary telerehabilitation.

Three trials [31,40,43] reported CAT (chronic obstructive pulmonary disease assessment test) scores at the end of the intervention. Pooled analysis showed no statistically significant difference between Tele-PR and CBPR (k=3; n=298, 18.0%; MD 0.24, 95% CI –8.44 to 8.92; *P=*.91; τ²=10.42; *I²*=89.1%; PI=–16.98 to 17.50; Figure 10).

**Figure 11.**
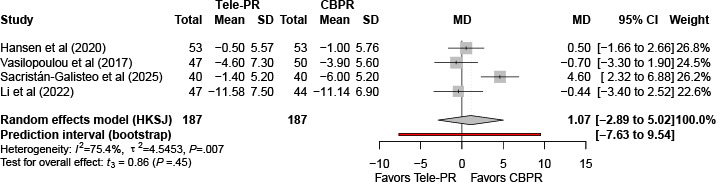
Forest plot of randomized controlled trials evaluating the effect of interventions on chronic obstructive pulmonary disease assessment test in patients with COPD at long-term follow-up (≥6 mo) [31,32,40,43]. The analysis was performed using a random effects model. CBPR: center-based pulmonary rehabilitation; HKSJ: Hartung-Knapp-Sidik-Jonkman; MD: mean difference; Tele-PR: pulmonary telerehabilitation.

At long-term follow-up [31,32,40,43], pooled analysis showed no statistically significant overall difference (k=4; n=374, 22.6%; MD 1.07, 95% CI –2.89 to 5.20; *P*=.45; τ²=4.54; *I²*=75.4%; PI=–7.63 to 9.54; Figure 11).

#### Chronic Respiratory Questionnaire–Dyspnea

**Figure 12.**
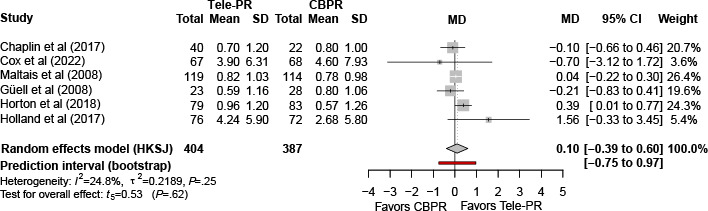
Forest plot of randomized controlled trials evaluating the effect of interventions on Chronic Respiratory Questionnaire–Dyspnea domain in patients with chronic obstructive pulmonary disease at the end of the intervention [27,33,36,37,39,41]. The analysis was performed using a random effects model. CBPR: center-based pulmonary rehabilitation; HKSJ: Hartung-Knapp-Sidik-Jonkman; MD: mean difference; Tele-PR: pulmonary telerehabilitation.

Six trials [27,33,36,37,39,41] reported Chronic Respiratory Questionnaire–Dyspnea outcomes at the end of the intervention. Pooled analysis showed no statistically significant difference between Tele-PR and CBPR (k=6; n=791, 47.7%; MD 0.10, 95% CI –0.39 to 0.60; *P=*.62; τ²=0.22; *I²*=24.8%; PI=–0.75 to 0.97; Figure 12).

**Figure 13.**
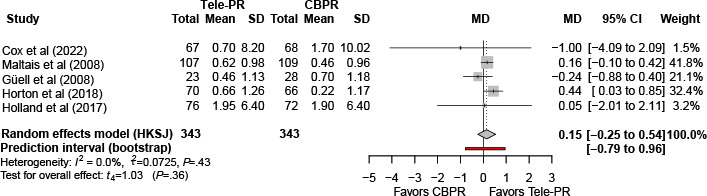
Forest plot of randomized controlled trials evaluating the effect of interventions on Chronic Respiratory Questionnaire–Dyspnea domain in patients with chronic obstructive pulmonary disease at long-term follow-up (≥6 mo) [33,36,37,39,41]. The analysis was performed using a random effects model. CBPR: center-based pulmonary rehabilitation; HKSJ: Hartung-Knapp-Sidik-Jonkman; MD: mean difference; Tele-PR: pulmonary telerehabilitation.

At long-term follow-up [33,36,37,39,41], pooled analysis also showed no statistically significant difference (k=5; n=686, 41.4%; MD 0.15, 95% CI –0.25 to 0.54; *P=*.34; τ²=0.07; *I²*=0%; PI=-0.79 to 0.96; Figure 13).

#### Hospital Anxiety and Depression Scale–Anxiety

**Figure 14.**
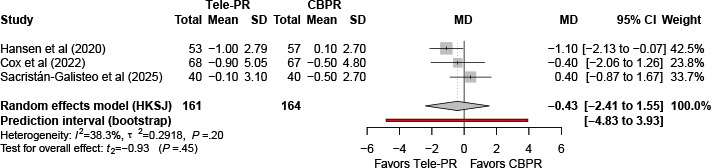
Forest plot of randomized controlled trials evaluating the effect of interventions on Hospital Anxiety and Depression Scale–Anxiety subscale in patients with chronic obstructive pulmonary disease at the end of intervention [31,33,43]. The analysis was performed using a random effects model. CBPR: center-based pulmonary rehabilitation; HKSJ: Hartung-Knapp-Sidik-Jonkman; MD: mean difference; Tele-PR: pulmonary telerehabilitation.

Three trials [31,33,43] reported Hospital Anxiety and Depression Scale–Anxiety outcomes at the end of the intervention. Pooled analysis showed no statistically significant difference between Tele-PR and CBPR (k=3; n=325, 19.6%; MD –0.43, 95% CI –2.41 to 1.55; *P=*.45; τ²=0.29; *I²*=38.3%; PI=–4.83 to 3.93; Figure 14).

**Figure 15.**
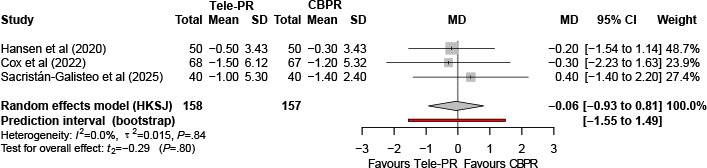
Forest plot of randomized controlled trials evaluating the effect of interventions on Hospital Anxiety and Depression Scale–Anxiety subscale in patients with chronic obstructive pulmonary disease at long-term follow-up (≥6 mo) [31,33,43]. The analysis was performed using a random effects model. CBPR: center-based pulmonary rehabilitation; HKSJ: Hartung-Knapp-Sidik-Jonkman; MD: mean difference; Tele-PR: pulmonary telerehabilitation.

At long-term follow-up [31,33,43], pooled analysis showed no statistically significant overall difference (Figure 15).

#### Hospital Anxiety and Depression Scale–Depression

**Figure 16.**
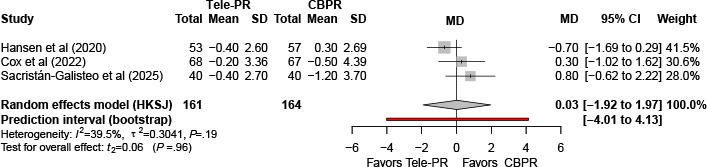
Forest plot of randomized controlled trials evaluating the effect of interventions on Hospital Anxiety and Depression Scale–Depression subscale in patients with chronic obstructive pulmonary disease at the end of intervention [31,33,43]. The analysis was performed using a random effects model. CBPR: center-based pulmonary rehabilitation; HKSJ: Hartung-Knapp-Sidik-Jonkman; MD: mean difference; Tele-PR: pulmonary telerehabilitation.

Three trials [31,33,43] reported Hospital Anxiety and Depression Scale–Depression outcomes at the end of the intervention. Pooled analysis showed no statistically significant difference between Tele-PR and CBPR (k=3; n=325, 19.6%; MD 0.03, 95% CI –1.92 to 1.97; *P=*.96; τ²=0.30; *I²*=39.5%; PI =–4.01 to 4.13; Figure 16).

**Figure 17.**
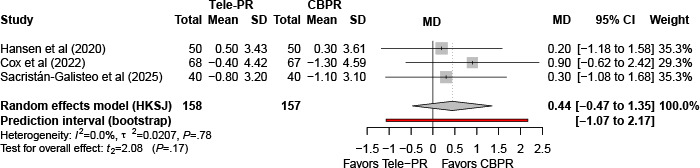
Forest plot of randomized controlled trials evaluating the effect of interventions on Hospital Anxiety and Depression Scale–Depression subscale in patients with chronic obstructive pulmonary disease at long-term follow-up (≥6 mo) [31,33,43]. The analysis was performed using a random effects model. CBPR: center-based pulmonary rehabilitation; HKSJ: Hartung-Knapp-Sidik-Jonkman; MD: mean difference; Tele-PR: pulmonary telerehabilitation.

At long-term follow-up [31,33,43], pooled analysis likewise showed no statistically significant overall difference (Figure 17).

#### Modified Medical Research Council Questionnaire

**Figure 18.**
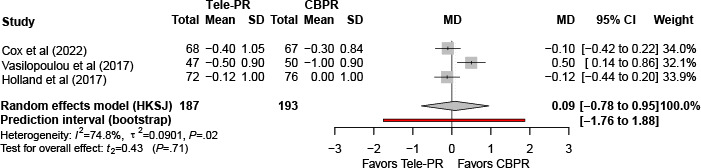
Forest plot of randomized controlled trials evaluating the effect of interventions on the modified Medical Research Council in patients with chronic obstructive pulmonary disease at the end of intervention [33,40,41]. The analysis was performed using a random effects model. CBPR: center-based pulmonary rehabilitation; HKSJ, Hartung-Knapp-Sidik-Jonkman; MD: mean difference; Tele-PR: pulmonary telerehabilitation.

Three trials [33,40,41] reported modified Medical Research Council questionnaire outcomes at the end of intervention. Pooled analysis showed no statistically significant difference between Tele-PR and CBPR (k=3; n=380, 22.9%; MD 0.09, 95% CI –0.78 to 0.95; *P=*.71; τ²=0.09; *I²*=74.8%; PI=–1.76 to 1.88; Figure 18).

**Figure 19.**
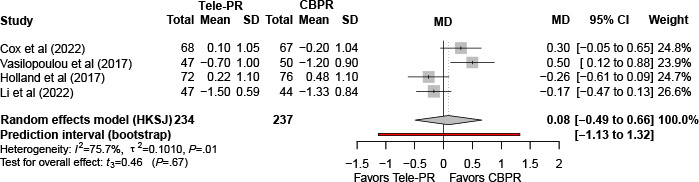
Forest plot of randomized controlled trials evaluating the effect of interventions on the modified Medical Research Council in patients with chronic obstructive pulmonary disease at long-term follow-up (≥6 mo) [32,33,40,41]. The analysis was performed using a random effects model. CBPR: center-based pulmonary rehabilitation; HKSJ: Hartung-Knapp-Sidik-Jonkman; MD: mean difference; Tele-PR: pulmonary telerehabilitation.

At long-term follow-up [32,33,40,41], pooled analysis showed no statistically significant overall difference (Figure 19).

#### Pulmonary Rehabilitation Adapted Index of Self-Efficacy

**Figure 20.**
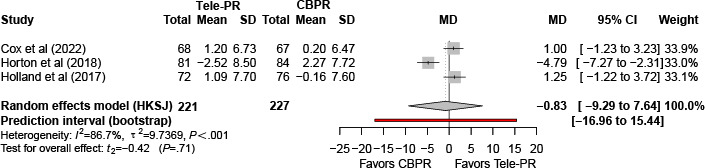
Forest plot of randomized controlled trials evaluating the effect of interventions on Pulmonary Rehabilitation Adapted Index of Self-Efficacy in patients with COPD at the end of intervention [33,39,41]. The analysis was performed using a random effects model. CBPR: center-based pulmonary rehabilitation; HKSJ: Hartung-Knapp-Sidik-Jonkman; MD: mean difference; Tele-PR: pulmonary telerehabilitation.

Three trials [33,39,41] reported Pulmonary Rehabilitation Adapted Index of Self-Efficacy outcomes at the end of the intervention. Pooled analysis showed no statistically significant difference between Tele-PR and CBPR (k=3; n=448, 27.0%; MD –0.83, 95% CI –9.29 to 7.64; *P=*.71; τ²=9.74; *I²*=86.7%; PI=–16.96 to 15.44; Figure 20).

**Figure 21.**
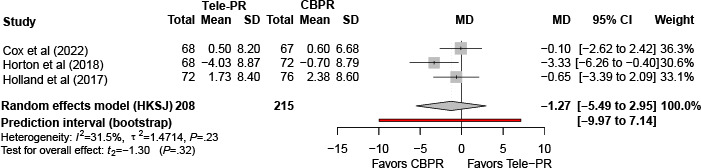
Forest plot of randomized controlled trials evaluating the effect of interventions on Pulmonary Rehabilitation Adapted Index of Self-Efficacy in patients with chronic obstructive pulmonary disease at long-term follow-up (≥6 mo) [33,39,41]. The analysis was performed using a random effects model. CBPR: center-based pulmonary rehabilitation; HKSJ: Hartung-Knapp-Sidik-Jonkman; MD: mean difference; Tele-PR: pulmonary telerehabilitation.

At long-term follow-up [33,39,41], pooled analysis likewise showed no statistically significant overall difference (Figure 21).

#### Dropout Rate

**Figure 22.**
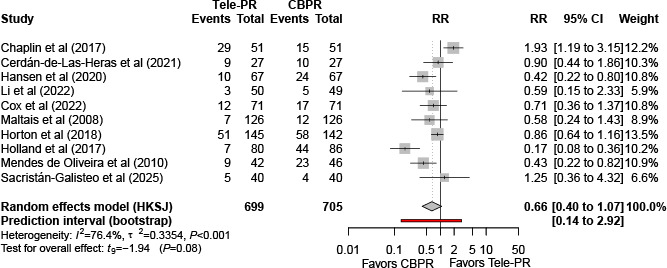
Forest plot of randomized controlled trials evaluating the effect of interventions assessing dropout rates in patients with chronic obstructive pulmonary disease at the end of intervention [27,29,31-33,36,39,41-43]. The analysis was performed using a random effects model. CBPR: center-based pulmonary rehabilitation; HKSJ: Hartung-Knapp-Sidik-Jonkman; RR: risk ratio; Tele-PR: pulmonary telerehabilitation.

Ten trials [27,29,31-33,36,39,41-43] reported end of intervention dropout data. Pooled random effects meta-analysis with HKSJ adjustment showed no statistically significant difference in dropout rate between Tele-PR and CBPR (k=10; n=1404, 84.7%; risk ratio 0.66, 95% CI 0.40-1.07; *P=*.08; τ²=0.33; *I²*=76.4%; PI=0.15-3.01; Figure 22).

Meta-regression analyses indicated that intervention duration, training intensity, publication year, baseline FEV_1_ (%), and economic status were not statistically significantly associated with dropout rate (all *P>*.05; Table 4 and Multimedia Appendix 7).

**Figure 23.**
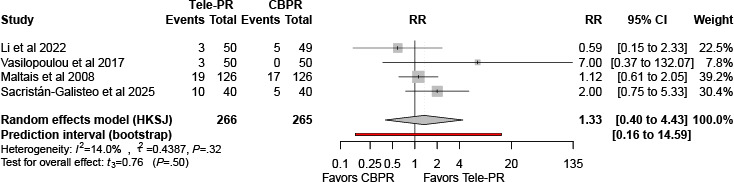
Forest plot of randomized controlled trials evaluating the effect of interventions assessing dropout rates in patients with chronic obstructive pulmonary disease at long-term follow-up (≥6 mo) [32,36,40,43]. The analysis was performed using a random effects model. CBPR: center-based pulmonary rehabilitation; HKSJ: Hartung-Knapp-Sidik-Jonkman; RR: risk ratio; Tele-PR: pulmonary telerehabilitation.

At long-term follow-up, pooled analysis of 4 studies [32,36,40,43] showed no statistically significant difference in dropout rate (Figure 23).

**Table 4.** Meta-regression results of examining covariates influencing end of intervention dropout rate.

Covariate	β coefficient (SE)	*t* (df)	*P* value
Intervention duration (weeks)	0.08 (0.12)	0.68 (8)	.52
Training intensity (medium vs high)	0.04 (0.55)	0.07 (8)	.94
Economic status (developing vs developed)	−0.38 (0.60)	−0.64 (8)	.54
Publication year	0.03 (0.05)	0.76 (8)	.47
Baseline FEV_1_^a^ (%)	0.02 (0.03)	0.85 (8)	.42

a FEV_1_: forced expiratory volume in 1 second.

#### Endurance Shuttle Walk Test

Two trials [27,39] Endurance Shuttle Walk Test outcomes. In a study by Horton et al [39], both home-based and center-based PR demonstrated clinically meaningful improvements exceeding the minimal clinically important difference (mean change 212 s vs 353 s, respectively). The between-group difference was −141 seconds (95% CI −252 to −31; *P*=.01). In a study by Chaplin et al [27], significant within-group improvements were observed in both the web-based (mean change 189 s) and conventional PR groups (mean change 185 s), with no significant between-group difference.

#### Willingness to Pay and Direct Costs

Willingness to pay did not differ meaningfully between Tele-PR and CBPR, with reported means of approximately US $175 to $176 and medians of US $83 to $100, and no statistically significant difference was detected (*P=*.98) [28]. In 1 study [41], the estimated cost of Tele-PR was AUD 298 (US $224), which was slightly lower than that of CBPR at AUD 312 (US $234), corresponding to a difference of AUD 14 (US $11).

#### Adverse Events

Eight (47.1%) [29,31,33,36,39-41,43] of 17 included trials reported adverse events or clinical safety outcomes, including serious adverse events, hospitalizations, acute exacerbations of COPD, or deaths. Among studies that explicitly monitored serious adverse events, no intervention-related serious adverse events were identified [31,33,36,39,41]. Event rates, where reported, were generally similar between telerehabilitation and center-based rehabilitation [31,33,36,40]. However, harms were not consistently prespecified or systematically reported across trials, and the scope and definitions of adverse events varied substantially.

### Subgroup Analysis

Subgroup analyses were conducted according to delivery models, supervision intensity, and supervision modality (Multimedia Appendix 7). For 6MWD at both the end of intervention and long-term follow-up (≥6 mo), no statistically significant subgroup differences were observed across delivery models, supervision intensity, or supervision modality (all *P*>.05), although moderate heterogeneity was noted in some low-technology HBPR strata. For daily steps and Chronic Respiratory Questionnaire–Dyspnea (CRQ-D) domain, no significant subgroup effects by delivery models were identified at either time point (all *P*>.05). In contrast, for the CAT, delivery models showed significant subgroup differences at both end of intervention and long-term follow-up (both *P*<.001), with digitally supported Tele-PR demonstrating small, nonsignificant changes, whereas low-technology HBPR showed worsening CAT scores in single-study strata. For the dropout rate, no significant subgroup differences were detected by delivery models or supervision modality (all *P*>.05), although heterogeneity was substantial in several subgroups. Overall, subgroup effects were generally limited, except for CAT, and several strata were constrained by small numbers of studies.

### Sensitivity Analysis and Publication Bias Test

Leave-one-out sensitivity analyses were conducted for all primary outcomes to assess the robustness of pooled estimates and to identify influential studies contributing to between-study heterogeneity (Multimedia Appendix 8). Overall, pooled estimates for several outcomes showed sensitivity to the exclusion of individual studies, indicating that some findings were influenced by specific trial characteristics.

For 6MWD, exclusion of a single telephone-based study [40] resulted in a shift of the pooled estimate toward CBPR (MD –8.63 m; *P=*.045), indicating that the overall effect estimate was sensitive to this study. For daily step counts, the marginally significant pooled effect was driven primarily by 1 study [35]; removal of this study rendered the pooled effect nonsignificant (*P=*.12) and substantially reduced heterogeneity.

For patient-reported outcomes, substantial heterogeneity in health status measured by the CAT was attributable to a single study [43]; exclusion of this study eliminated heterogeneity and attenuated the pooled effect. Similarly, heterogeneity in self-efficacy outcomes was largely driven by 1 study [39], and removal of this study reduced between-study variance and altered the pooled estimate.

In contrast, pooled estimates for modified Medical Research Council Dyspnea score, depressive symptoms, and dropout rate were robust to leave-one-out analyses. Although individual studies [34,41] showed effects in opposite directions for dropout, the overall conclusion of no statistically significant difference between groups remained unchanged across all sensitivity analyses.

**Figure 24.**
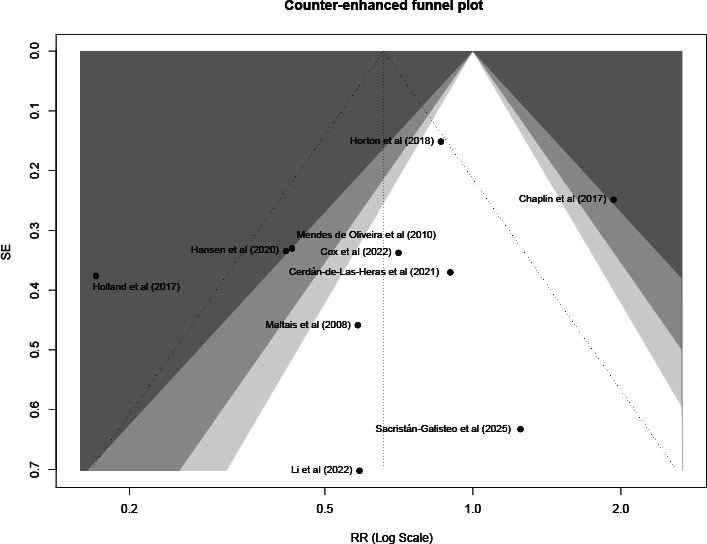
Contour-enhanced funnel plot for the assessment of publication bias in dropout rates [27,29,31-33,36,39,41-43]. Each dot represents an individual study included in the meta-analysis. The vertical axis represents the SE (a measure of study precision), and the horizontal axis represents the risk ratio (log scale). The shaded regions indicate statistical significance levels (*P*<.10, *P*<.05, and *P*<.01), while the white area corresponds to nonsignificant results (*P*>.10).

Visual inspection of the funnel plot for dropout rate revealed a relatively symmetrical distribution of studies (Figure 24). This was confirmed by Peters test, which showed no evidence of significant publication bias or small-study effects (*t(8)*=.43; *P*=.68). However, given the limited number of included studies, the results of publication bias tests should be interpreted cautiously.

### GRADE Assessment

The certainty of evidence ranged from moderate to very low across outcomes. Evidence for 6MWD was rated as low certainty and was downgraded by 1 level for risk of bias because blinding was not feasible and attrition occurred in several trials. Evidence for dropout rate was rated as very low certainty and was downgraded for risk of bias, substantial inconsistency, and imprecision, reflected by wide CIs and PI*s*. Health status outcomes (CAT, SGRQ, and Chronic Respiratory Questionnaire–Dyspnea) were rated as low certainty and were downgraded for inconsistency, likely related to heterogeneous intervention modalities, and for imprecision. These certainty ratings should temper interpretation of pooled estimates and underscore variability in real-world effects. The certainty of evidence assessed using the GRADE approach is summarized in Table 5.

**Table 5.** Summary of findings and certainty of evidence (GRADE) for tele-pulmonary rehabilitation compared with center-based pulmonary rehabilitation.

Certainty assessment							Patients, n		Effect		Certainty	Importance
Studies (n)	Study design	Risk of bias	Inconsistency	Indirectness	Imprecision	Other considerations	Intervention	Comparison	Relative (95% CI)	Absolute (95% CI)		
6-Minute walk distance (end of intervention)												
9	Randomized trials	Serious^a^	Serious^b^	Not serious	Not serious	None	476	474	—^c^	MD^d^ −5.37 m (−15.68 to 4.95)	⨁⨁◯◯Low^a^^,b^	Critical
6-Minute walk distance (long-term follow-up [≥6 mo])												
9	Randomized trials	Serious^a^	Serious^e^	Not serious	Serious^f^	None	475	473	—	MD 2.97 m (−12.22 to 18.17)	⨁◯◯◯Very low^a^^,e^	Critical
Dropout rate (end of intervention)												
10	Randomized trials	Serious^a^	Serious^g^	Not serious	Serious^h^	None	142/699 (20.3%)	212/705 (30.1%)	RR^i^ 0.66 (0.40 to 1.07)	102 fewer per 1000 (from −180 to 21)	⨁◯◯◯Very low^a^^,g,h^	Critical
Dropout rate (long-term follow-up [≥6 mo])												
4	Randomized trials	Serious^a^	Not serious	Not serious	Very serious^j^	None	35/266 (13.2%)	27/265 (10.2%)	RR 1.33 (0.40 to 4.43)	34 more per 1000 (from 61 to 349)	⨁◯◯◯Very low^a^^,j^	Critical
COPD assessment test (end of intervention)												
3	Randomized trials	Serious^a^	Serious^k^	Not serious	Serious^l^	None	146	152	—	MD 0.24 (−8.44 to 8.92)	⨁◯◯◯Very low^a^^,k,l^	Important
COPD assessment test (long-term follow-up [≥6 mo])												
4	Randomized trials	Serious^a^	Serious^m^	Not serious	Serious^n^	None	187	187	—	MD 1.07 (−2.89 to 5.2)	⨁◯◯◯Very low^a^^,m,n^	Important
Daily steps (end of intervention)												
5	Randomized trials	Serious^a^	Serious^o^	Not serious	Serious^p^	None	119	134	—	MD 4.97 (−1.84 to 11.78 )	⨁◯◯◯Very low^a^^,o,p^	Important
Daily steps (long-term follow-up [≥6 mo])												
3	Randomized trials	Serious^a^	Not serious	Not serious	Very serious^q^	None	69	73	—	MD −1.01 (−6.27 to 4.2)	⨁◯◯◯Very low^a^^,q^	Important
St. George’s Respiratory Questionnaire (end of intervention)												
3	Randomized trials	Serious^a^	Serious^r^	Not serious	Serious^s^	None	184	178	—	MD 0.45 (−7.11 to 8 )	⨁◯◯◯Very low^a^^,r,s^	Important
St. George’s Respiratory Questionnaire (long-term follow-up [≥6 mo])												
3	Randomized trials	Serious^a^	Not serious	Not serious	Serious^t^	None	169	167	—	MD −0.01 (−5.08 to 5.05)	⨁⨁◯◯Low^a^^,t^	Important
Chronic Respiratory Questionnaire–Dyspnea (end of intervention)												
6	Randomized trials	Serious^a^	Not serious	Not serious	Not serious	None	404	387	—	MD 0.1 (−0.39 to 0.6)	⨁⨁⨁◯Moderate^a^	Important
Chronic Respiratory Questionnaire–Dyspnea (long-term follow-up [≥6 mo])												
5	Randomized trials	Serious^a^	Not serious	Not serious	Serious^u^	None	343	343	—	MD 0.15 (−0.25 to 0.54)	⨁⨁◯◯Low^a^^,u^	Important
Hospital Anxiety and Depression Scale–Anxiety (end of intervention)												
3	Randomized trials	Serious^a^	Not serious	Not serious	Serious^v^	None	161	164	—	MD −0.43 (−2.41 to 1.55)	⨁⨁◯◯Low^a^^,v^	Important
Hospital Anxiety and Depression Scale–Anxiety (long-term follow-up [≥6 mo])												
3	Randomized trials	Serious^a^	Not serious	Not serious	Serious^w^	None	158	157	—	MD −0.06 (−0.93 to 0.81 **)**	⨁⨁◯◯Low^a^^,w^	Important
Hospital Anxiety and Depression Scale–Depression (end of intervention)												
3	Randomized trials	Serious^a^	Not serious	Not serious	Serious^v^	None	161	164	—	MD 0.03 (−1.92 to 1.97**)**	⨁⨁◯◯Low^a^^,v^	Important
Hospital Anxiety and Depression Scale–Depression (long-term follow-up [≥6 mo])												
3	Randomized trials	Serious^a^	Not serious	Not serious	Serious^x^	None	158	157	—	MD **0.44** (−0.47 to 1.35)	⨁⨁◯◯Low^a^^,x^	Important
Modified Medical Research Council Questionnaire (end of intervention)												
3	Randomized trials	Serious^a^	Serious^y^	Not serious	Not serious	None	187	193	—	MD 0.09 (−0.78 to 0.95**)**	⨁⨁◯◯Low^a^^,y^	Important
Modified Medical Research Council Questionnaire (long-term follow-up [≥6 mo])												
4	Randomized trials	Serious^a^	Serious^z^	Not serious	Serious^aa^	None	234	237	—	MD 0.08 (−0.49 to 0.6)	⨁◯◯◯Very low^a^^,z,aa^	Important
PR Adapted Index of Self-Efficacy (end of intervention)												
3	Randomized trials	Serious^a^	Serious^ab^	Not serious	Serious^ac^	None	221	227	—	MD −0.83 (−9.29 to 7.6)	⨁◯◯◯Very low^a^^,ab,ac^	Important
PR Adapted Index of Self-Efficacy (long-term follow-up [≥6 mo])												
3	Randomized trials	Serious^a^	Not serious	Not serious	Serious^ad^	None	206	215	—	MD −1.27 (−5.49 to 2.95)	⨁⨁◯◯Low^a^^,ad^	Important

a Most studies had a high risk of performance bias due to lack of blinding of participants and personnel, which is inherent to exercise interventions.

b Downgraded for inconsistency due to model-level variability and prediction intervals spanning clinically important benefit and harm across settings.

c Not applicable.

d MD: mean difference.

e Downgraded 1 level due to moderate heterogeneity (*I*2=44.5%) and variability in long-term adherence across studies.

f Although the CI crosses the line of no effect, it excludes the minimal clinically important difference of 30 m, suggesting no clinically meaningful difference.

g Downgraded 1 level due to extreme heterogeneity (*I*2=86%) with conflicting directions of effect.

h Downgraded 1 level due to wide CIs (0.40-1.07) crossing the line of no effect.

i RR: risk ratio.

j Downgraded 2 levels due to very wide CIs that cross the line of no effect and include potential for both substantial benefit and substantial harm.

k Substantial heterogeneity was observed across studies, with inconsistent effect estimates across intervention models.

l CIs include both benefit and no effect and do not consistently exceed the minimal clinically important difference.

m Downgraded 1 level due to high heterogeneity (*I*2=75%) with conflicting directions of effect across studies.

n Downgraded 1 level due to wide CIs that cross the line of no effect and include the minimal clinically important difference of 2 points for potential harm.

o Although statistical heterogeneity was low, effect estimates were sensitive to individual studies and varied across intervention models.

p CIs include both increased and decreased daily step counts and do not exclude no effect.

q Downgraded 2 levels due to very small sample size (n=142, below the optimal information size) and wide CIs that cross the line of no effect.

r inconsistent effect estimates across different intervention models.

s CIs include both benefit and no effect and do not consistently exceed the minimal clinically important difference.

t Downgraded 1 level due to CIs crossing the line of no effect and including the minimal clinically important difference of 4 units in both directions.

u Downgraded 1 level due to CIs crossing the line of no effect and including the minimal clinically important difference of 0.5 points in both directions.

v CIs included both benefit and no effect, and the total sample size was limited, resulting in imprecision.

w Downgraded 1 level due to limited sample size (n=315, below optimal information size) and CIs crossing the line of no effect.

x Downgraded 1 level due to limited sample size (n=315, below optimal information size) and CIs crossing the line of no effect.

y Substantial heterogeneity was observed across studies, with inconsistent effect estimates.

z Downgraded 1 level due to high heterogeneity with conflicting directions of effect.

aa Downgraded 1 level due to wide CIs that cross the line of no effect and include the minimal clinically important difference of 0.5 points in both directions.

ab Substantial heterogeneity was observed across studies, with highly variable effect estimates depending on intervention modality.

ac CIs were wide and crossed the line of no effect, and no established minimal clinically important difference is available for Pulmonary Rehabilitation Adapted Index of Self-Efficacy.

ad Downgraded 1 level due to wide CIs crossing the line of no effect.

## Discussion

### Principal Findings

This systematic review and meta-analysis evaluated the relative effectiveness of Tele-PR compared to CBPR in patients with COPD. Overall, no statistically significant differences were observed at the end of the intervention across key outcomes, including functional capacity, dyspnea, and health-related quality of life. These findings indicate that, on average, Tele-PR may achieve short-term clinical effects similar to those of CBPR when implemented under structured and well-defined conditions. However, subsequent subgroup analyses and investigations of heterogeneity suggest that Tele-PR should not be regarded as a single homogeneous intervention [33]. Substantial variation was observed in effect consistency and stability across different remote delivery models, particularly with respect to supervision intensity and interaction modality. Accordingly, interpretations of “equivalence” between Tele-PR and CBPR based solely on pooled average effects should be made with caution, as such summaries may obscure meaningful differences across implementation contexts [19].

### Long-Term Efficacy and Maintenance Rehabilitation

In this analysis, differences in outcomes between rehabilitation modalities diminished during postintervention follow-up, indicating a time-dependent convergence. This pattern suggests that, regardless of delivery format, remote and center-based PR programs face challenges in sustaining long-term benefits [3]. Several factors may contribute to this attenuation of effects, although these potential explanatory variables were not directly assessed in this meta-analysis. First, adherence to regular exercise training often declines after structured supervision ends, particularly when ongoing support is limited [44,45]. Second, longer follow-up periods may include seasonal variations, acute exacerbations, or intercurrent illnesses, which can disrupt training continuity and negatively impact functional outcomes [46,47]. Third, many existing Tele-PR programs primarily focus on exercise training, while systematic reinforcement of relapse prevention and long-term self-management skills remains relatively underdeveloped [48]. Importantly, this convergence of outcomes does not imply that early rehabilitation effects lack clinical significance. Rather, it underscores the need to conceptualize PR—whether delivered remotely or in person—as an ongoing process that requires sustained behavioral and educational support, rather than as a time-limited intervention. This perspective aligns with the concept of maintenance rehabilitation emphasized in recent clinical guidelines and supports the future integration of educational components, behavioral maintenance strategies, and digital platforms to improve long-term effectiveness [49-51].

### Physiological Mechanisms and Safety Profile

The included studies predominantly enrolled patients with moderate-to-severe COPD, a population in whom home-based training has traditionally raised safety concerns [3]. Evidence suggests that, under appropriate screening and standardized implementation, Tele-PR was not associated with increased short-term serious adverse events and demonstrated a safety profile comparable to that of CBPR [31,33,36,39,41]. Functional improvements likely extend beyond aerobic enhancement, as PR improves respiratory mechanics and ventilatory efficiency [14,52-54]. Although physiological markers were not directly synthesized, dyspnea improvements in Tele-PR suggest overlapping mechanisms with conventional PR [55]. Emerging evidence of improved gas exchange in hypercapnic patients undergoing PR further supports integrating refined physiological monitoring in higher-risk subgroups [56].

### Supervision Modalities and Psychological Safety

An important observation from this review is that supervision intensity may contribute to heterogeneity in the effectiveness of remote rehabilitation. Subgroup analyses [31,33] indicated that synchronous, video-supervised programs demonstrated lower statistical heterogeneity in functional capacity outcomes, whereas low-supervision or asynchronous approaches exhibited greater variability. These findings raise the possibility that, beyond training dose alone, real-time interaction may support psychological safety, perceived support, and confidence during exercise, which in turn may influence engagement and adherence [48,57]. Conversely, insufficient supervision in home-based programs may heighten concerns regarding exercise-related risk and limit participation [58,59]. However, these interpretations should be approached cautiously. Given the exploratory nature of subgroup analyses and the limited number of studies within specific supervision categories, the independent contributions of psychological factors, adherence, and training intensity cannot be clearly disentangled. Future trials should prospectively incorporate measures of psychological safety, self-efficacy, and fear of exercise to clarify the pathways through which supervision modalities influence outcomes.

### Comparison With Standard Care

The relative effectiveness of Tele-PR was closely linked to the characteristics of the comparator intervention. When compared to lower-intensity programs or usual care, Tele-PR was more likely to demonstrate a favorable relative effect [60]; however, when compared to high-intensity, face-to-face supervised rehabilitation, these advantages were substantially attenuated [43]. These findings suggest that conclusions regarding the equivalence of Tele-PR should be interpreted as context dependent rather than as intrinsic or universally applicable [61]. Accordingly, Tele-PR is best viewed as complementary to, rather than as a universal substitute for, high-quality center-based rehabilitation, with optimal use determined by patient characteristics, resource availability, and service capacity [62].

### Barriers to Implementation and Digital Literacy

A notable gap was observed between improvements in functional capacity and changes in daily physical activity. Although gains in exercise capacity were observed under certain remote rehabilitation models, corresponding increases in daily step counts were not consistently demonstrated and were sensitive to the influence of individual studies [35]. This pattern suggests that functional improvement alone may be insufficient to produce sustained behavioral change [63]. Future Tele-PR strategies may therefore need to incorporate structured behavioral interventions alongside monitoring and feedback mechanisms. Digital literacy and technology access remain implementation barriers [64,65], and simpler, low-technology models with strong human support may achieve higher adherence in certain populations [41]. Platforms should prioritize usability and minimize cognitive burden to avoid undermining effectiveness [66,67].

### Cost-Effectiveness and Economic Considerations

Although this review primarily focused on clinical outcomes, economic considerations are critical for the sustainable implementation of remote rehabilitation. Available evidence [32,40,68,69] suggests that Tele-PR is broadly comparable to CBPR in terms of direct program costs and may offer potential long-term economic benefits by improving accessibility and intervention completion rates, consistent with findings from recent cost-utility evaluations of digital therapeutics for PR. However, cost-effectiveness evidence remains heterogeneous [70], underscoring the need for standardized economic evaluations in future trials.

### Innovation and Contribution

Previous systematic reviews of PR and telerehabilitation in COPD have largely focused on estimating average between-group differences, reporting minimal clinically important difference achievement, or comparing remote and center-based delivery formats [71,72], while foundational Cochrane work established the overall efficacy of PR compared with usual care [73]. Few syntheses have systematically examined supervision-related heterogeneity or incorporated PIs to estimate expected effect ranges across settings. We observed substantial variation in program structure, supervision models, and comparator intensity, complicating the interpretation of pooled averages. By integrating supervision-based stratification with PI-informed interpretation, this review provides a context-sensitive framework for evaluating Tele-PR relative to CBPR. To our knowledge, it is among the first to frame Tele-PR equivalence as context dependent rather than model intrinsic, supporting tailored implementation in real-world health care systems.

### Limitations

Several limitations should be acknowledged. First, most included studies were nonblinded, and implementation bias could not be fully avoided. Second, the number of long-term follow-up studies was limited, resulting in insufficient statistical power for certain long-term outcome analyses. Third, definitions of “usual care” and rehabilitation intensity varied across studies, potentially introducing residual confounding. In addition, several subgroup analyses were based on a small number of studies and should therefore be interpreted as exploratory rather than definitive, particularly where a subgroup was informed by a single trial. The term “noninferiority” as used in this study refers to the overall distribution of effect directions and their 95% CIs, rather than a prespecified noninferiority margin; thus, it should be interpreted as indicating clinical equivalence rather than constituting a strict statistical test of noninferiority. Consistent with the PRISMA-S reporting framework, the omission of trial registry and gray literature searches may have resulted in the underrepresentation of ongoing, unpublished, or null-result trials, potentially contributing to residual publication bias despite comprehensive database coverage. Furthermore, the small number of studies available for several outcomes precluded formal risk of bias–based sensitivity analyses, which should be taken into account when assessing the robustness of the findings. The clinical implications outlined earlier are primarily based on evidence of moderate to low certainty and should be applied cautiously in real-world settings, with due consideration of local resources, patient characteristics, and operational conditions. Subgroup and meta-regression analyses were exploratory and intended to generate hypotheses rather than confirm model superiority.

### Conclusions

Distinct from prior reviews that treated Tele-PR as a homogeneous intervention, we stratified remote programs by supervision intensity and delivery models and proposed a structured “supervision gradient” framework to interpret model-dependent consistency of effects, with practical implications for designing Tele-PR as a supervision-structured service incorporating real-time oversight and long-term behavioral maintenance support. Tele-PR is a feasible option for delivering PR to adults with COPD and, on average, yields short-term clinical outcomes comparable to those of CBPR. As effects varied across studies and the certainty of the evidence was low to very low, implementation should be context aware and model specific. Programs incorporating structured real-time professional supervision appear to produce more consistent outcomes, whereas low-technology HBPR may require enhanced behavioral support to achieve stable symptom control and improvements in daily activity levels. Longer-term maintenance remains a challenge, indicating that remote rehabilitation should be designed as an ongoing service rather than a time-limited intervention. Accordingly, Tele-PR may be particularly valuable for expanding access to PR and addressing existing accessibility gaps, while CBPR remains essential for patients who require close in-person supervision or complex multidisciplinary care.

## Supplementary material

10.2196/80500 Multimedia Appendix 1 Changes to preregistered protocol.

10.2196/80500 Multimedia Appendix 2 Full electronic search strategies for all databases.

10.2196/80500 Multimedia Appendix 3 Reasons for exclusion of full-text articles.

10.2196/80500 Multimedia Appendix 4 Audit of study independence linkage of multiple reports via trial registration numbers and strategy.

10.2196/80500 Multimedia Appendix 5 Key intervention strategies and supervision models.

10.2196/80500 Multimedia Appendix 6 Risk of bias assessment of included randomized controlled trials.

10.2196/80500 Multimedia Appendix 7 Subgroup analyses and meta-regression plots.

10.2196/80500 Multimedia Appendix 8 Leave-one-out sensitivity analyses for all primary outcomes.

10.2196/80500 Checklist 1 PRISMA 2020, PRISMA 2020 for abstracts, and PRISMA-S checklists.

## Abbreviations

CAT: chronic obstructive pulmonary disease assessment test
CBPR: center-based pulmonary rehabilitation
COPD: chronic obstructive pulmonary disease
GAIDeT: Generative Artificial Intelligence Delegation Taxonomy
Gen-AI: generative artificial intelligence
GRADE: Grading of Recommendations, Assessment, Development, and Evaluation
HBPR: home-based pulmonary rehabilitation
HKSJ: Hartung-Knapp-Sidik-Jonkman
MD: mean difference
PI: prediction interval
PICOS: population, intervention, comparator, outcomes, and study design
PR: pulmonary rehabilitation
PRISMA: Preferred Reporting Items for Systematic Reviews and Meta-Analyses
RCT: randomized controlled trial
SGRQ: St George’s Respiratory Questionnaire
Tele-PR: pulmonary telerehabilitation

## References

[1] GBD 2016 Occupational Chronic Respiratory Risk Factors Collaborators (2020). Global and regional burden of chronic respiratory disease in 2016 arising from non-infectious airborne occupational exposures: a systematic analysis for the Global Burden of Disease Study 2016. Occup Environ Med.

[2] Rothnie KJ, Müllerová H, Smeeth L, Quint JK (2018). Natural history of chronic obstructive pulmonary disease exacerbations in a general practice-based population with chronic obstructive pulmonary disease. Am J Respir Crit Care Med.

[3] Spruit MA, Singh SJ, Garvey C (2013). An official American Thoracic Society/European Respiratory Society statement: key concepts and advances in pulmonary rehabilitation. Am J Respir Crit Care Med.

[4] Rochester CL, Alison JA, Carlin B (2023). Pulmonary rehabilitation for adults with chronic respiratory disease: an Official American Thoracic Society Clinical Practice Guideline. Am J Respir Crit Care Med.

[5] Alison JA, McKeough ZJ, Johnston K (2017). Australian and New Zealand Pulmonary Rehabilitation Guidelines. Respirology.

[6] Spitzer KA, Stefan MS, Priya A (2019). Participation in pulmonary rehabilitation after hospitalization for chronic obstructive pulmonary disease among Medicare beneficiaries. Ann Am Thorac Soc.

[7] Desveaux L, Janaudis-Ferreira T, Goldstein R, Brooks D (2015). An international comparison of pulmonary rehabilitation: a systematic review. COPD.

[8] Sharma A, Harrington RA, McClellan MB (2018). Using digital health technology to better generate evidence and deliver evidence-based care. J Am Coll Cardiol.

[9] Bhavnani SP, Parakh K, Atreja A (2017). 2017 Roadmap for Innovation-ACC Health Policy Statement on healthcare transformation in the era of digital health, big data, and precision health: a report of the American College of Cardiology Task Force on health policy statements and systems of care. J Am Coll Cardiol.

[10] Zhuang M, Hassan II, W Ahmad WMA (2025). Effectiveness of digital health interventions for chronic obstructive pulmonary disease: systematic review and meta-analysis. J Med Internet Res.

[11] Cox NS, McDonald C, Burge AT, Hill CJ, Bondarenko J, Holland AE (2025). Comparison of clinically meaningful improvements after center-based and home-based telerehabilitation in people with COPD. Chest.

[12] Sánchez-Romero EA, García-Barredo-Restegui T, Martínez-Rolando L (2025). Addressing post-COVID-19 musculoskeletal symptoms through pulmonary rehabilitation with an evidence-based eHealth education tool: preliminary results from a pilot randomized controlled clinical trial. Medicine (Abingdon).

[13] Martínez-Pozas O, Corbellini C, Cuenca-Zaldívar JN, Meléndez-Oliva É, Sinatti P, Sánchez Romero EA (2024). Effectiveness of telerehabilitation versus face-to-face pulmonary rehabilitation on physical function and quality of life in people with post COVID-19 condition: a systematic review and network meta-analysis. Eur J Phys Rehabil Med.

[14] Martínez-Pozas O, Meléndez-Oliva E, Rolando LM, Rico JAQ, Corbellini C, Sánchez Romero EA (2024). The pulmonary rehabilitation effect on long covid-19 syndrome: a systematic review and meta-analysis. Physiother Res Int.

[15] Page MJ, McKenzie JE, Bossuyt PM (2021). The PRISMA 2020 statement: an updated guideline for reporting systematic reviews. BMJ.

[16] Rethlefsen ML, Kirtley S, Waffenschmidt S (2021). PRISMA-S: an extension to the PRISMA statement for reporting literature searches in systematic reviews. J Med Libr Assoc.

[17] McHugh ML (2012). Interrater reliability: the kappa statistic. Biochem Med (Zagreb).

[18] IntHout J, Ioannidis JPA, Borm GF (2014). The Hartung-Knapp-Sidik-Jonkman method for random effects meta-analysis is straightforward and considerably outperforms the standard DerSimonian-Laird method. BMC Med Res Methodol.

[19] Nagashima K, Noma H, Furukawa TA (2019). Prediction intervals for random-effects meta-analysis: a confidence distribution approach. Stat Methods Med Res.

[20] Borenstein M (2023). How to understand and report heterogeneity in a meta-analysis: the difference between I-squared and prediction intervals. Integr Med Res.

[21] Sterne JAC, Sutton AJ, Ioannidis JPA (2011). Recommendations for examining and interpreting funnel plot asymmetry in meta-analyses of randomised controlled trials. BMJ.

[22] Peters JL, Sutton AJ, Jones DR, Abrams KR, Rushton L (2006). Comparison of two methods to detect publication bias in meta-analysis. JAMA.

[23] Thompson SG, Higgins JPT (2002). How should meta-regression analyses be undertaken and interpreted?. Stat Med.

[24] Senn SJ (2009). Overstating the evidence: double counting in meta-analysis and related problems. BMC Med Res Methodol.

[25] Sterne JAC, Savović J, Page MJ (2019). RoB 2: a revised tool for assessing risk of bias in randomised trials. BMJ.

[26] What is GRADE?. GRADE.

[27] Chaplin E, Hewitt S, Apps L (2017). Interactive web-based pulmonary rehabilitation programme: a randomised controlled feasibility trial. BMJ Open.

[28] Burge AT, Holland AE, McDonald CF (2021). “Willingness to pay”: the value attributed to program location by pulmonary rehabilitation participants. COPD: Journal of Chronic Obstructive Pulmonary Disease.

[29] Cerdán-de-Las-Heras J, Balbino F, Løkke A, Catalán-Matamoros D, Hilberg O, Bendstrup E (2021). Effect of a new tele-rehabilitation program versus standard rehabilitation in patients with chronic obstructive pulmonary disease. J Clin Med.

[30] Hansen H, Torre A, Kallemose T, Ulrik CS, Godtfredsen NS (2023). Pulmonary telerehabilitation vs. conventional pulmonary rehabilitation - a secondary responder analysis. Thorax.

[31] Hansen H, Bieler T, Beyer N (2020). Supervised pulmonary tele-rehabilitation versus pulmonary rehabilitation in severe COPD: a randomised multicentre trial. Thorax.

[32] Li Y, Qian H, Yu K, Huang Y (2022). The long-term maintenance effect of remote pulmonary rehabilitation via social media in copd: a randomized controlled trial. Int J Chron Obstruct Pulmon Dis.

[33] Cox NS, McDonald CF, Mahal A (2022). Telerehabilitation for chronic respiratory disease: a randomised controlled equivalence trial. Thorax.

[34] Chaplin E, Barnes A, Newby C, Houchen-Wolloff L, Singh SJ (2022). Comparison of the impact of conventional and web-based pulmonary rehabilitation on physical activity in patients with chronic obstructive pulmonary disease: exploratory feasibility study. JMIR Rehabil Assist Technol.

[35] Horton EJ, Ruksenaite J, Mitchell K, Sewell L, Newby C, Singh SJ (2021). A comparison of physical activity between home-based and centre-based pulmonary rehabilitation: a randomised controlled secondary analysis. Front Rehabil Sci.

[36] Maltais F, Bourbeau J, Shapiro S (2008). Effects of home-based pulmonary rehabilitation in patients with chronic obstructive pulmonary disease: a randomized trial. Ann Intern Med.

[37] Güell MR, de Lucas P, Gáldiz JB (2008). Home vs hospital-based pulmonary rehabilitation for patients with chronic obstructive pulmonary disease: a Spanish multicenter trial. Arch Bronconeumol.

[38] Lahham A, McDonald CF, Mahal A (2019). Participation in physical activity during center and home-based pulmonary rehabilitation for people with COPD: a secondary analysis of a randomized controlled trial. J Cardiopulm Rehabil Prev.

[39] Horton EJ, Mitchell KE, Johnson-Warrington V (2018). Comparison of a structured home-based rehabilitation programme with conventional supervised pulmonary rehabilitation: a randomised non-inferiority trial. Thorax.

[40] Vasilopoulou M, Papaioannou AI, Kaltsakas G (2017). Home-based maintenance tele-rehabilitation reduces the risk for acute exacerbations of COPD, hospitalisations and emergency department visits. Eur Respir J.

[41] Holland AE, Mahal A, Hill CJ (2017). Home-based rehabilitation for COPD using minimal resources: a randomised, controlled equivalence trial. Thorax.

[42] Mendes de Oliveira JC, Studart Leitão Filho FS, Malosa Sampaio LM (2010). Outpatient vs. home-based pulmonary rehabilitation in COPD: a randomized controlled trial. Multidiscip Respir Med.

[43] Sacristán-Galisteo C, Del Corral T, Gómez-Pesquera C (2025). Long-term effects of face-to-face supervision versus telephone supervision during a community-based pulmonary rehabilitation programme in people with COPD using minimal equipment: a randomized controlled trial. BMC Prim Care.

[44] Jansons PS, Haines TP, O’Brien L (2017). Interventions to achieve ongoing exercise adherence for adults with chronic health conditions who have completed a supervised exercise program: systematic review and meta-analysis. Clin Rehabil.

[45] Duregon F, Quinto G, Vecchiato M (2025). From theory into practice: insights from a real-world implementation model for tailored exercise prescription in chronic diseases. BMC Sports Sci Med Rehabil.

[46] Josa-Culleré A, Basagaña X, Koch S (2024). Short-term effects of air pollution and weather on physical activity in patients with chronic obstructive pulmonary disease (COPD). Environ Res.

[47] Donaldson GC, Goldring JJ, Wedzicha JA (2012). Influence of season on exacerbation characteristics in patients with COPD. Chest.

[48] Olsen M, Nielsen C, Emme C, Godtfredsen NS, Hansen H (2025). Why we keep going: a qualitative longitudinal study of the motivation and engagement among patients with COPD during a long-term Danish tele-rehabilitation program. Patient Prefer Adherence.

[49] (2025). Global strategy for the diagnosis, management, and prevention of chronic obstructive lung disease 2025 report. https://goldcopd.org/2025-gold-report/.

[50] Holland AE, Cox NS, Houchen-Wolloff L (2021). Defining modern pulmonary rehabilitation. An official American Thoracic Society Workshop Report. Ann Am Thorac Soc.

[51] Rochester CL, Vogiatzis I, Holland AE (2015). An official American Thoracic Society/European Respiratory Society Policy Statement: enhancing implementation, use, and delivery of pulmonary rehabilitation. Am J Respir Crit Care Med.

[52] Nacarato D, Sardeli AV, Mariano LO, Chacon-Mikahil MPT (2024). Cardiovascular telerehabilitation improves functional capacity, cardiorespiratory fitness and quality of life in older adults: a systematic review and meta-analysis. J Telemed Telecare.

[53] Richardson CR, Franklin B, Moy ML, Jackson EA (2019). Advances in rehabilitation for chronic diseases: improving health outcomes and function. BMJ.

[54] Corbellini C, Rossino E, Massaccesi R (2022). Improvements in perimeter thoracic mobility on patients with COPD after pulmonary rehabilitation: a case series. ELECTRON J GEN MED.

[55] Chung C, Kim AR, Kang DY (2025). Clinical efficacy of smartphone app-based pulmonary rehabilitation in chronic respiratory diseases: randomized controlled and feasibility trials. J Med Internet Res.

[56] Corbellini C, Tavella S, Gugliotta E, Zampese S, Sanchez-Romero EA, Villafane J Hypercapnia and functional improvements during pulmonary rehabilitation.

[57] Chan SWC (2021). Chronic disease management, self-efficacy and quality of life. J Nurs Res.

[58] Fukuta Y, Arizono S, Tanaka S (2023). Effects of real-time remote cardiac rehabilitation on exercise capacity and quality of life: a quasi-randomised controlled trial. BMC Geriatr.

[59] Saka S, Gurses HN, Bayram M (2021). Effect of inspiratory muscle training on dyspnea-related kinesiophobia in chronic obstructive pulmonary disease: a randomized controlled trial. Complement Ther Clin Pract.

[60] Sakunrag I, Boontha N, Boonpattharatthiti K, Dhippayom T (2025). Impact of tele-pulmonary rehabilitation in patients with chronic obstructive disease: a systematic review and network meta-analysis. Telemed J E Health.

[61] Burge AT, Cox NS, Abramson MJ, Holland AE (2020). Interventions for promoting physical activity in people with chronic obstructive pulmonary disease (COPD). Cochrane Database Syst Rev.

[62] Ward TJC, Latimer L, Daynes E (2025). Impact of pulmonary rehabilitation programme design on effectiveness in COPD: a systematic review and component network meta-analysis. EClinicalMedicine.

[63] Spruit MA, Pitta F, McAuley E, ZuWallack RL, Nici L (2015). Pulmonary rehabilitation and physical activity in patients with chronic obstructive pulmonary disease. Am J Respir Crit Care Med.

[64] Choi NG, Dinitto DM (2013). The digital divide among low-income homebound older adults: internet use patterns, eHealth literacy, and attitudes toward computer/Internet use. J Med Internet Res.

[65] Wilson J, Heinsch M, Betts D, Booth D, Kay-Lambkin F (2021). Barriers and facilitators to the use of e-health by older adults: a scoping review. BMC Public Health.

[66] Bentley CL, Powell L, Potter S (2020). The use of a smartphone app and an activity tracker to promote physical activity in the management of chronic obstructive pulmonary disease: randomized controlled feasibility study. JMIR Mhealth Uhealth.

[67] Chengyu Z, Xueyan H, Ying F (2024). Research on disease management of chronic disease patients based on digital therapeutics: a scoping review. Digit Health.

[68] Zanaboni P, Dinesen B, Hoaas H (2023). Long-term telerehabilitation or unsupervised training at home for patients with chronic obstructive pulmonary disease: a randomized controlled trial. Am J Respir Crit Care Med.

[69] Park H, Jang M, Kim D (2025). Cost-utility analysis and value-based pricing of digital therapeutics for pulmonary rehabilitation in chronic respiratory disease: economic evaluation based on a randomized controlled trial. J Med Internet Res.

[70] Liu S, Zhao Q, Li W, Zhao X, Li K (2021). The cost-effectiveness of pulmonary rehabilitation for COPD in different settings: a systematic review. Appl Health Econ Health Policy.

[71] Bondarenko J, Dal Corso S, Dillon MP (2024). Clinically important changes and adverse events with centre-based or home-based pulmonary rehabilitation in chronic respiratory disease: a systematic review and meta-analysis. Chron Respir Dis.

[72] Zhu J, Kikuchi Y, Nishimura S, Shibata K (2025). Physiological and psychological therapeutic effects of telerehabilitation treatment on patients with chronic obstructive pulmonary disease: a systematic review and meta-analysis. J Phys Ther Sci.

[73] McCarthy B, Casey D, Devane D, Murphy K, Murphy E, Lacasse Y (2015). Pulmonary rehabilitation for chronic obstructive pulmonary disease. Cochrane Database Syst Rev.

[74] Suchikova Y, Tsybuliak N, Teixeira da Silva JA, Nazarovets S (2026). GAIDeT (Generative AI Delegation Taxonomy): a taxonomy for humans to delegate tasks to generative artificial intelligence in scientific research and publishing. Account Res.

