# Supplementary material for: Comparing Pulmonary Telerehabilitation and Center-Based Pulmonary Rehabilitation for Effectiveness and Adherence in Chronic Obstructive Pulmonary Disease: Systematic Review and Meta-Analysis of Randomized Controlled Trials
Source: J Med Internet Res. 2026 Apr 17;28:e80500. doi: 10.2196/80500 (PMC13089800; doi:10.2196/80500)
Supplement: Multimedia Appendix 1 [file jmir-v28-e80500-s001.docx]

**Multimedia Appendix 1. Changes to pre-registered protocol**

In our PROSPERO-registered protocol (CRD42025633252), we stated

1. First, although the protocol originally planned to search both English- and Chinese-language databases, the final review was restricted to four major English-language databases—PubMed, Embase, the Cochrane Central Register of Controlled Trials (CENTRAL), and the Web of Science Core Collection. Preliminary scoping searches revealed substantial overlap in the literature indexed across Chinese and English databases; critically, no additional randomized controlled trials meeting our prespecified eligibility criteria were identified beyond those retrieved from the English-language sources. Given this redundancy the decision was made prior to full-text screening to focus exclusively on English-language databases to maximize methodological rigor and resource efficiency.
2. Second, while the protocol specified use of the PEDro scale for risk-of-bias assessment, the Cochrane Risk of Bias 2 (RoB 2) tool was adopted in the final review. RoB 2 is widely endorsed as the gold-standard instrument for evaluating bias in randomized trials, offering a domain-based, algorithm-driven assessment that improves transparency, reproducibility, and sensitivity to trial-specific design features—advantages not afforded by the PEDro scale. This update aligns the review with current best practices recommended by the Cochrane Handbook and the GRADE Working Group.
3. Third, the statistical analysis plan was refined to enhance inferential robustness. Whereas the protocol proposed conventional random-effects meta-analysis using RevMan, all analyses were conducted in R (version 4.3.3) using the *meta* package, implementing Hartung–Knapp–Sidik–Jonkman (HKSJ)–adjusted random-effects models. The HKSJ adjustment provides more accurate confidence intervals—particularly under conditions of sparse data (e.g., ≤5 studies per subgroup) or high heterogeneity—and reduces type I error inflation compared with standard DerSimonian–Laird methods. Furthermore, prediction intervals were reported alongside summary effect estimates for all primary outcomes to quantify the anticipated range of true effects across diverse clinical settings—a key advancement for evidence translation and implementation.
4. Fourth, although the protocol listed a broad set of secondary outcomes, several—including hospital readmission rates, COPD exacerbation frequency, and health-related quality of life subscales—were not subjected to meta-analysis due to either insufficient reporting (e.g., zero studies providing usable event data) or high methodological heterogeneity (e.g., inconsistent definitions or measurement tools). These outcomes are therefore presented descriptively in the results and discussed contextually in the narrative synthesis.
5. Finally, subgroup analyses were refined during the review to explicitly contrast two conceptually distinct home-based rehabilitation modalities: (1) digitally supported Tele-PR, characterized by real-time or asynchronous digital monitoring, feedback, or coaching; and (2) low-technology HBPR, delivered without digital infrastructure (e.g., paper-based programs, telephone support only). Supervision intensity was also pre-specified as a key conceptual moderator. These subgroup comparisons remain exploratory and hypothesis-generating, designed to inform clinical decision-making and future trial design rather than to test prespecified causal hypotheses. All deviations were documented prior to data synthesis and approved by the review team based on methodological justification.
