# Supplementary material for: Comparing Pulmonary Telerehabilitation and Center-Based Pulmonary Rehabilitation for Effectiveness and Adherence in Chronic Obstructive Pulmonary Disease: Systematic Review and Meta-Analysis of Randomized Controlled Trials
Source: J Med Internet Res. 2026 Apr 17;28:e80500. doi: 10.2196/80500 (PMC13089800; doi:10.2196/80500)
Supplement: Multimedia Appendix 2 [file jmir-v28-e80500-s002.docx]

**Search strategy used in PubMed** Search date: December 10, 2025

| **Step** | **Search Query (Concept)** | **Results** |
| --- | --- | --- |
| **#1** | **Concept: COPD (Population)**  ("Pulmonary Disease, Chronic Obstructive"[MeSH Terms] OR "pulmonary disease, chronic obstructive"[Title/Abstract] OR "chronic obstructive pulmonary disease"[Title/Abstract] OR "COPD"[Title/Abstract] OR "chronic obstructive airways disease"[Title/Abstract] OR "chronic obstructive lung disease"[Title/Abstract]) | 121,534 |
| **#2** | **Concept: Telerehabilitation**  ("Telerehabilitation"[MeSH Terms] OR "Telerehabilitation"[Title/Abstract] OR "tele rehabilitation"[Title/Abstract] OR "remote rehabilitation"[Title/Abstract] OR "virtual rehabilitation"[Title/Abstract] OR "rehabilitation virtual"[Title/Abstract] OR "e-rehabilitation"[Title/Abstract]) | 4,310 |
| **#3** | **Concept: Telemedicine/mHealth**  ("Telemedicine"[MeSH Terms] OR "Telemedicine"[Title/Abstract] OR "tele referral"[Title/Abstract] OR "virtual medicine"[Title/Abstract] OR "tele intensive care"[Title/Abstract] OR "tele icu"[Title/Abstract] OR "mobile health"[Title/Abstract] OR "mHealth"[Title/Abstract] OR "Telehealth"[Title/Abstract] OR "eHealth"[Title/Abstract]) | 94,790 |
| **#4** | **Concept: Virtual Reality**  ("Virtual Reality"[MeSH Terms] OR "virtual reality"[Title/Abstract] OR "reality virtual"[Title/Abstract] OR "educational virtual realities"[Title/Abstract] OR "virtual environments"[Title/Abstract] OR "virtuality"[Title/Abstract] OR "virtualization"[Title/Abstract]) | 27,309 |
| **#5** | **Concept: Video Games/Computer-assisted**  ("Home-based"[Title/Abstract] OR "Videogame"[Title/Abstract] OR "computergame"[Title/Abstract] OR "television game"[Title/Abstract] OR "tv games"[Title/Abstract] OR "computerized treatment"[Title/Abstract] OR "computerized training"[Title/Abstract] OR "computer-assisted"[Title/Abstract]) | 53,979 |
| **#6** | **Combined Interventions**  #2 OR #3 OR #4 OR #5 | 173,719 |
| **#7** | **Final Search**  #1 AND #6 | **1,816** |

**Search strategy used in Web of Science Core Collection** Search date: December 10, 2025

| **Step** | **Search Query (Topic Search)** | **Results** |
| --- | --- | --- |
| **#1** | **Concept: COPD (Population)**  TS=("pulmonary disease, chronic obstructive" OR "chronic obstructive pulmonary disease" OR "COPD" OR "chronic obstructive airways disease" OR "chronic obstructive lung disease" OR ("pulmonary" AND "disease" AND "chronic" AND "obstructive")) | 121,458 |
| **#2** | **Concept: Telemedicine/mHealth**  TS=("Telemedicine" OR "tele referral" OR "virtual medicine" OR "medicine virtual" OR "tele intensive care" OR "tele icu" OR "mobile health" OR "health mobile" OR "mHealth" OR "Telehealth" OR "EHealth") | 91,041 |
| **#3** | **Concept: Virtual Reality & Education**  TS=("instructed" OR "instructing" OR "instructional" OR "instructions" OR "instructive" OR "instructively" OR "instructiveness" OR "instructs" OR "teaching" OR "instruction") AND ("virtual realities" OR "Realities" OR "Reality" OR "instructional virtual") | 85,623 |
| **#4** | **Concept: Video Games/Home-based**  TS=("Home-based" OR "Videogame" OR "computergame" OR "television game" OR "tv games" OR "computerized treatment" OR "computerized training" OR "computer-assisted") | 74,411 |
| **#5** | **Concept: Telenursing & Others**  TS=("Telenursing" OR "virtual nursing") OR (TS=(("lung" OR "pulmonary") AND "hypertension") AND TS=("Telerehabilitation" OR "tele rehabilitation" OR "remote rehabilitation" OR "virtual rehabilitation" OR "e-rehabilitation")) | 13* |
| **#6** | **Combined Interventions**  #2 OR #3 OR #4 OR #5 | 247,320 |
| **#7** | **Final Search**  #1 AND #6 | **1,970** |

**Search strategy used in The Cochrane Library** Search date: December 10, 2025

| **ID** | **Search Query** | **Results** |
| --- | --- | --- |
| **#1** | **Concept: COPD (Population)**  ("Pulmonary Disease, Chronic Obstructive" OR "Chronic Obstructive Pulmonary Disease" OR "Chronic Obstructive Airways Disease" OR ("Pulmonary" AND "Disease" AND "Chronic" AND "Obstructive") OR ("Chronic" AND "Obstructive" AND "Airways" AND "Disease")) | 26,099 |
| **#2** | **Concept: Telerehabilitation**  ("Telerehabilitation" OR "Tele Rehabilitation" OR "Remote Rehabilitation" OR "Rehabilitation Remote" OR "Virtual Rehabilitation" OR "Rehabilitation Virtual" OR "E-Rehabilitation") | 2,445 |
| **#3** | **Concept: Telenursing**  ("Telenursing" OR "Virtual Nursing") | 384 |
| **#4** | **Concept: Telemedicine/mHealth**  ("Telemedicine" OR "Tele Referral" OR "Virtual Medicine" OR "Medicine Virtual" OR "Tele Intensive Care" OR "Tele ICU" OR "Mobile Health" OR "Health Mobile" OR "mHealth" OR "Telehealth" OR "EHealth") | 17,502 |
| **#5** | **Concept: Virtual Reality**  ("Virtual Reality" OR "Reality Virtual" OR "Educational Virtual Realities" OR "Virtual Environments" OR "Virtuality" OR "Virtualization" OR "Virtualized" OR "Virtualizing" OR "Virtuals" OR ("Realities" AND "Educational") OR ("Teaching" AND "Virtual Realities")) | 39,287 |
| **#6** | **Concept: Video Games/Computer-assisted**  ("Home-based" OR "Videogame" OR "Computergame" OR "Television Game" OR "TV Games" OR "Computerized Treatment" OR "Computerized Training" OR "Computer-assisted") | 34,862 |
| **#7** | **Combined Interventions**  #2 OR #3 OR #4 OR #5 OR #6 | 89,392 |
| **#8** | **Final Search**  #1 AND #7 | **1,609** |

**Search strategy used in Embase** Search date: December 10, 2025

| **Step** | **Search Query (Embase Syntax)** | **Results** |
| --- | --- | --- |
| **#1** | **Concept: COPD (Population)**  'pulmonary disease, chronic obstructive'/exp OR 'pulmonary disease, chronic obstructive' OR ('pulmonary':ab,ti AND 'disease':ab,ti AND 'chronic':ab,ti AND 'obstructive':ab,ti) OR 'chronic obstructive pulmonary disease':ab,ti OR ('chronic':ab,ti AND 'obstructive':ab,ti AND 'airways':ab,ti AND 'disease':ab,ti) OR 'chronic obstructive airways disease':ab,ti | 233,473 |
| **#2** | **Concept: Telerehabilitation**  'telerehabilitation'/exp OR 'telerehabilitation':ab,ti OR 'tele rehabilitation':ab,ti OR 'remote rehabilitation':ab,ti OR 'rehabilitation remote':ab,ti OR 'virtual rehabilitation':ab,ti OR 'rehabilitation virtual':ab,ti OR 'e-rehabilitation':ab,ti | 6,274 |
| **#3** | **Concept: Telemedicine/mHealth**  'telemedicine'/exp OR 'telemedicine':ab,ti OR 'tele referral':ab,ti OR 'virtual medicine':ab,ti OR 'medicine virtual':ab,ti OR 'tele intensive care':ab,ti OR 'tele icu':ab,ti OR 'mobile health':ab,ti OR 'health mobile':ab,ti OR 'mhealth':ab,ti OR 'telehealth':ab,ti OR 'ehealth':ab,ti | 131,251 |
| **#4** | **Concept: Virtual Reality & Education**  'virtual reality'/exp OR 'virtual reality':ab,ti OR 'reality virtual':ab,ti OR 'educational virtual realities':ab,ti OR (('realities':ab,ti OR 'reality':ab,ti) AND 'educational virtual':ab,ti) OR 'virtuality':ab,ti OR 'virtualization':ab,ti OR (('teaching'/exp OR 'teaching':ab,ti OR 'instruction':ab,ti) AND 'virtual realities':ab,ti) | 46,062 |
| **#5** | **Concept: Video Games/Computer-assisted**  'home-based':ab,ti OR 'videogame':ab,ti OR 'computergame':ab,ti OR 'television game':ab,ti OR 'tv games':ab,ti OR 'computerized treatment':ab,ti OR 'computerized training':ab,ti OR 'computer-assisted':ab,ti | 67,875 |
| **#6** | **Combined Interventions**  #2 OR #3 OR #4 OR #5 | 239,761 |
| **#7** | **Final Search**  #1 AND #6 | **3,452** |
