# Supplementary material for: Comparing Pulmonary Telerehabilitation and Center-Based Pulmonary Rehabilitation for Effectiveness and Adherence in Chronic Obstructive Pulmonary Disease: Systematic Review and Meta-Analysis of Randomized Controlled Trials
Source: J Med Internet Res. 2026 Apr 17;28:e80500. doi: 10.2196/80500 (PMC13089800; doi:10.2196/80500)
Supplement: Multimedia Appendix 3 [file jmir-v28-e80500-s003.docx]

****Multimedia Appendix 3. Reasons for exclusion of full-text articles.****

| **ID** | **Study** | **Exclusion Reason** |
| --- | --- | --- |
| 1 | Alwakeel, A. J. (2020) | Not RCT |
| 2 | Candy, S. (2023) | Not RCT |
| 3 | Bhatt, S. P. (2021) | Not RCT |
| 4 | Lewis, A. (2021) | Not RCT |
| 5 | Pehlivan, E. (2019) | Not RCT |
| 6 | Knox, L. (n.d.) | Not RCT |
| 7 | Candemir, I. (2019) | Not RCT |
| 8 | Bryant, M. S. (2019) | Not RCT |
| 9 | Bhatt, S. P. (2019) | Not RCT |
| 10 | Paneroni, M. (2015) | Not RCT |
| 11 | Jansen-Kosterink, S. (2015) | Not RCT |
| 12 | Debeaumont, D. (2015) | Not RCT |
| 13 | Jolly, E. (2014) | Not RCT |
| 14 | Grosbois, J. M. (2013) | Not RCT |
| 15 | Tousignant, M. (2012) | Not RCT |
| 16 | Doke, Prakash (2025) | Not RCT |
| 17 | Wootton, S. (2024) | Not RCT |
| 18 | Spencer, Lissa (2024) | Not RCT |
| 19 | Ashworth, N. L. (2009) | Comparator Mismatch |
| 20 | Bourbeau, J. (2019) | Comparator Mismatch |
| 21 | Tupper, O. D. (2018) | Comparator Mismatch |
| 22 | Soriano, J. B. (2018) | Comparator Mismatch |
| 23 | Janaudis-Ferreira, T. (2018) | Comparator Mismatch |
| 24 | Zwerink, M. (2016) | Comparator Mismatch |
| 25 | Franke, K. J. (2016) | Comparator Mismatch |
| 26 | Blumenthal, J. A. (2014) | Comparator Mismatch |
| 27 | Dias, F. D. (2013) | Comparator Mismatch |
| 28 | Sönnerfors, Pernilla (2025) | Comparator Mismatch |
| 29 | Knight-Davidson, P. (2025) | Comparator Mismatch |
| 30 | Huang, Xueying (2025) | Comparator Mismatch |
| 31 | Glynn, Lisa (2025) | Comparator Mismatch |
| 32 | Racodon, Michaël (2024) | Comparator Mismatch |
| 33 | Maltais, F. (2005) | Study Protocol |
| 34 | Xin, Hongxia (2024) | Study Protocol |
| 35 | Karim, Rania (2025) | Study Protocol |
| 36 | Anonymous | Study Protocol |
| 37 | Shi, Xin-Yu (2024) | Study Protocol |
| 38 | Varhan, Berrak (2025) | Study Protocol |
| 39 | Bonnevie, Tristan (2025) | Study Protocol |
| 40 | Dowman, L. (2022) | Conference Abstract |
| 41 | Hajizadeh, N. (2023) | Conference Abstract |
| 42 | De Las Heras, J. C. (2020) | Conference Abstract |
| 43 | Cox, N. (2020) | Conference Abstract |
| 44 | Rassouli, F. (2018) | Conference Abstrac |
| 45 | Widyastuti, K. (2017) | Conference Abstract |
| 46 | Kaliaraju, D. (2017) | Conference Abstract |
| 47 | Drover, H. (2025) | Conference Abstract |
| 48 | Rutherford, H. (2024) | Conference Abstract |
| 49 | Cavalheri, V. (2022) | Critically Appraised Paper |
| 50 | Maltais, F. (2009) | Commentary |
| 51 | Gu, W. (2022) | Review |
| 52 | Sun, W., et al. | Review |
| 53 | Ellis, James (2025) | Review/Service Eval |
| 54 | Cox, Narelle S. (2025) | Secondary Analysis |
