# Supplementary material for: Comparing Pulmonary Telerehabilitation and Center-Based Pulmonary Rehabilitation for Effectiveness and Adherence in Chronic Obstructive Pulmonary Disease: Systematic Review and Meta-Analysis of Randomized Controlled Trials
Source: J Med Internet Res. 2026 Apr 17;28:e80500. doi: 10.2196/80500 (PMC13089800; doi:10.2196/80500)
Supplement: Multimedia Appendix 4 [file jmir-v28-e80500-s004.docx]

| **Multimedia Appendix 4. Audit of study independence: Linkage of multiple reports via trial registration numbers and strategy for data extraction** | | | | |
| --- | --- | --- | --- | --- |
| **Included Reports (Citations)** | | **Trial Registration No.** | **Study Relationship** | **Data Extraction Strategy** |
| Hansen et al. | 1. Hansen et al. (2020) [Main]  2. Hansen et al. (2023) [Long-term] | NCT02667171 | Primary efficacy report and long-term follow-up of the same RCT. | Time-point Separation: Data from Hansen (2020) were used *only* for post-intervention analysis. Data from Hansen (2023) were used *only* for 12-month follow-up analysis. No overlap in any single meta-analysis. |
| Horton et al. | 1. Horton et al. (2018) [Main]  2. Horton et al. (2021) [Secondary] | ISRCTN81189044 | Secondary analysis of the same RCT focusing on physical activity. | Outcome Separation: Main clinical outcomes (6MWD, CRQ) were extracted from Horton (2018). Physical activity data (Steps) were extracted from Horton (2021). |
| Holland et al. | 1. Holland et al. (2017) [Main]  2. Lahham et al. (2019) [Secondary] | NCT01423227 | Secondary analysis of the same RCT focusing on physical activity behavior. | Outcome Separation: 6MWD and symptoms were extracted from Holland (2017). Objective physical activity metrics (MVPA, Steps) were extracted from Lahham (2019). |
| Chaplin et al. | 1. Chaplin et al. (2017) [Feasibility]  2. Chaplin et al. (2022) [PA Report] | ISRCTN03142263 | Feasibility trial and subsequent detailed report on physical activity. | Priority Selection: Efficacy data were primarily extracted from the main feasibility study (2017). The 2022 report was verified as the same cohort and not treated as a new study. |
| Li et al. | 1. Li et al. (2022) [Three-arm RCT] | ChiCTR1900021320 | Multi-arm trial: (A) Tele-PR vs. (B) Hospital-PR vs. (C) Usual Care. | Arm Exclusion: Only Group A (Tele-PR) and Group B (Hospital-PR) were included. Group C (Usual Care) was excluded to prevent double-counting of the control group in multiple pairwise comparisons. |
