# Supplementary material for: Comparing Pulmonary Telerehabilitation and Center-Based Pulmonary Rehabilitation for Effectiveness and Adherence in Chronic Obstructive Pulmonary Disease: Systematic Review and Meta-Analysis of Randomized Controlled Trials
Source: J Med Internet Res. 2026 Apr 17;28:e80500. doi: 10.2196/80500 (PMC13089800; doi:10.2196/80500)
Supplement: Multimedia Appendix 5 [file jmir-v28-e80500-s005.docx]

| ****Multimedia Appendix 5. Key Intervention Strategies and Supervision Models**** | | | | |
| --- | --- | --- | --- | --- |
| ****Study**** | ****Tele-PR Program Name**** | ****Exercise Components****  ****(Content)**** | ****Exercise Prescription****  ****(Dose/Intensity/Frequency)**** | ****Supervision & Support Model**** |
| Chaplin et al. (2017) | SPACE for COPD (Web-based) | Aerobic (walking) + Strength + Education + Symptom diaries | Freq: Daily  Int: Walking at 85% baseline (ISWT); Strength at BORG 13-15 | Asynchronous: Remote monitoring via website + Weekly email/phone contact. |
| Holland et al. (2017) | Home-based PR | Aerobic (walking) + Strength (bands/weights) + Health coaching | Freq: Daily (walking) + 3x/week (strength)  Int: BORG 3-4 (Moderate) | Minimal: 1 initial home visit + 7 weekly telephone calls from physiotherapist. |
| Horton et al. (2018) | SPACE for COPD (Manual) | Aerobic (walking) + Strength + Education Manual | Freq: Daily  Int: BORG 3-4 | Unsupervised: Self-managed with manual + 2 telephone calls (Wk 2 & 4). |
| Hansen et al. (2020) | Pulmonary Tele-rehabilitation (PTR) | Aerobic (cycling/walking) + Strength + Inspiratory muscle training | Freq: 3x/week (60 min/session)  Int: High intensity (BORG 4-6) | Synchronous: Real-time videoconferencing with physiotherapist (group based). |
| Maltais et al. (2008) | Self-monitored Home PR | Aerobic (cycling) + Strength + Stretching | Freq: 3x/week  Int: 60-80% peak work rate | Remote: Initial home visit setup + Weekly telephone calls. |
| Vasilopoulou et al. (2017) | Home-based Tele-rehabilitation | Cycling + Strength + Monitoring (SpO2, HR) | Freq: Daily (30 min)  Int: BORG dyspnea < 5 | Asynchronous: Data upload 3-4x/week + Weekly feedback calls. |
| Sacristán-Galisteo (2025) | Telephone Supervision (TS) | Aerobic (walking/cycling) + Strength + Breathing exercises | Freq: 3x/week  Int: Aerobic (BORG 12-14); Strength (OMNI 5-7) | Remote: Weekly telephone calls (15-20 min) to adjust intensity & motivate. |
| Li et al. (2022) | WeChat-based PR | Aerobic (walking/stairs) + Strength + Breathing + Tai Chi | Freq: 3-5x/week  Int: BORG 12-14 (Target HR 60-80%) | Remote: Daily interaction via WeChat group (uploads & feedback). |
| Cox et al. (2021) | Virtual Group Tele-PR | Aerobic + Strength + Education | Freq: 2x/week  Int: BORG 3-4 | Synchronous: Real-time group videoconferencing (via tablet). |
| Cerdán-de-las-Heras (2022) | VAPA (App + Sensor) | Aerobic (step test) + Strength (elastics) + Breathing | Freq: Daily recommended  Int: AI-driven biofeedback adjustment | Hybrid: Autonomous AI supervision (real-time feedback) + Therapist checks. |
| Burge et al. (2021) | Low-tech Home-based | Aerobic + Strength + Motivational Interviewing | Freq: 5x/week (walking)  Int: BORG 3-4 | Minimal: 1 Home visit + 7 weekly telephone calls. |
| Chaplin et al. (2022) | Web-based PR (SPACE) | Aerobic + Strength + Education | Freq: Daily  Int: BORG 3-4 | Asynchronous: Online progress monitoring + Weekly contact. |
| Güell et al. (2008) | Unsupervised Home PR | Aerobic (walking) + Strength (upper/lower) + Breathing | Freq: Daily (walking)  Int: 70% max speed | Unsupervised: Instructions at start, then self-managed. |
| Lahham et al. (2019) | Home-based PR | Aerobic (walking) + Strength | Freq: 3-5x/week  Int: BORG 3-4 | Indirect: Initial supervision then weekly calls. |
| Mendes de Oliveira (2010) | At-home rehabilitation | Aerobic (walking) + Strength | Freq: 3x/week  Int: 80% peak speed | Unsupervised: Initial training at clinic + Telephone follow-up. |
| Horton et al. (2021) | Manual-supported PR | Aerobic + Strength | Freq: Daily  Int: BORG 3-4 | Minimal: Same as Horton 2018 (Manual + 2 calls). |

|  |
| --- |

**Abbreviations:** **BORG**: Borg Rating of Perceived Exertion Scale; **HR**: Heart Rate; **ISWT**: Incremental Shuttle Walk Test; **OMNI-RES**: Omni Resistance Exercise Scale; **SpO_2_**: Oxygen Saturation.
