# Supplementary material for: Comparing Pulmonary Telerehabilitation and Center-Based Pulmonary Rehabilitation for Effectiveness and Adherence in Chronic Obstructive Pulmonary Disease: Systematic Review and Meta-Analysis of Randomized Controlled Trials
Source: J Med Internet Res. 2026 Apr 17;28:e80500. doi: 10.2196/80500 (PMC13089800; doi:10.2196/80500)
Supplement: Multimedia Appendix 6 [file jmir-v28-e80500-s006.docx]

****Multimedia Appendix 6. Risk of bias assessment of included randomized controlled trials.****

| ****Study**** | ****Randomization Process**** | ****Deviations from Intended Interventions**** | ****Missing Outcome Data**** | ****Measurement of the Outcome**** | ****Selection of the Reported Result**** | ****Overall Risk of Bias**** |
| --- | --- | --- | --- | --- | --- | --- |
| **Chaplin et al. 2017** | **Low Risk:** Randomization was done using a third-party program, ensuring allocation concealment. Baseline characteristics were balanced. | **Some Concerns:** Participants were aware of the intervention, and analysis was done using completers instead of ITT. High dropout rate (57%) could introduce bias. | **High Risk:** High dropout rate (57%) in the intervention group, likely skewing the results in favor of the intervention. Exclusion of dropouts (who had worse baseline health) likely biases the effect estimate. | **Low Risk:** Objective outcome measures with blinded assessors. | **Low Risk:** All outcomes were reported according to protocol, no selective reporting. | **High Risk:** The high dropout rate and exclusion of "worst cases" likely introduce bias, overestimating the intervention's effect. |
| **Burge et al. 2021** | **Low Risk:** Randomization and allocation concealment were appropriately performed. | **Low Risk:** No significant deviations from the intended interventions. | **Low Risk:** Missing data (15%) was not related to baseline characteristics or outcomes, minimizing bias. | **Low Risk:** The outcome (WTP) was measured using a standardized method. | **Low Risk:** All outcomes were reported as per the protocol, with no selective reporting bias. | **Low Risk:** The study design and methods were rigorous, ensuring low bias across domains. |
| **Cerdán-de-las-Heras 2022** | **Some Concerns:** Randomization was performed, but significant baseline differences (age, SGRQ) were found, affecting group comparability. | **Low Risk:** No significant deviations from intended interventions. | **Some Concerns:** A high dropout rate (~35%) could compromise the robustness of the data, despite ITT analysis. | **Low Risk:** Objective, standardized outcome measures. | **Some Concerns:** High dropout and baseline imbalances could influence results, though all expected outcomes were reported. | **Some Concerns:** Significant baseline differences and high dropout rate raise concerns about the reliability of the results. |
| **Hansen et al., 2023** | **Low Risk:** Randomization and allocation concealment were properly executed, with no significant issues found. | **Some Concerns:** There were minor deviations in how the intervention was applied, which could introduce inconsistencies in the results, although these did not drastically impact the study's findings. | **Some Concerns:** The study reported 13% missing data in the short-term (10 weeks), which is borderline. For the long-term (62 weeks), missing data increased to 44%, which is very high and could substantially affect the reliability of the results. | **Low Risk:** Outcome measures were objective and standardized, minimizing measurement bias. | **Low Risk:** All outcomes were reported according to the pre-specified protocol, with no evidence of selective reporting. | **Some Concerns:** While randomization and outcome measurement were adequately performed, the high rate of missing data—especially in the long-term follow-up—raises concerns about the validity of the results. The high dropout rate may lead to biases and thus, the overall risk of bias is rated as "Some Concerns". |
| **Hansen et al., 2020** | **Low Risk:** Randomization and allocation concealment were properly executed, with no significant issues found. | **Low Risk:** No significant deviations from the intended interventions. | **High Risk:** The dropout rate in the control group (36%) was significantly higher than the experimental group (15%), introducing potential bias. Missing data is likely non-random (MNAR), as it is associated with outcomes like poor motor skills and severe illness. Despite using a mixed model to adjust, the missingness could still result in bias. | **Low Risk:** Objective and standardized outcome measures were used, minimizing measurement bias. | **Low Risk:** All pre‑specified outcomes were reported in full, with no evidence of selective reporting. | **High Risk:** The disproportionate dropout rate, especially in the control group, introduces significant bias. This could lead to overestimation or underestimation of the effectiveness of the intervention. |
| **Li et al., 2022** | **Low Risk:** Randomization was performed correctly, with no significant baseline differences. | **Some Concerns:** The study used a per‑protocol analysis, which could introduce bias. | **Low Risk:** Missing data was minimal (3.4%), and the analysis did not appear to be affected by it. | **Low Risk:** Objective outcome measures, though evaluators were not blinded. | **Low Risk:** All outcomes were reported as per protocol. | **Some Concerns:** The per-protocol analysis and lack of blinding increase the risk of bias. |
| **Cox et al., 2021** | **Low Risk:** Randomization was performed correctly, and allocation concealment was well‑handled. | **Low Risk:** No significant deviations from intended interventions. | **Low Risk:** Missing data (5%) was minimal, and evidence suggested it did not affect the results. | **Low Risk:** Objective outcome measures with blinded assessors. | **Low Risk:** All outcomes were reported fully and transparently. | **Low Risk:** Low bias across all domains, leading to a low overall risk of bias. |
| **Chaplin et al. 2022** | **Some Concerns:** Randomization was done, but only 39% of the participants were analyzed (per‑protocol analysis), leading to attrition bias. | **High Risk:** High dropout rate (61%) and exclusion of participants led to survival bias, favoring the intervention. | **High Risk:** Analysis based on completers introduces bias as dropouts were likely to have worse health. | **Low Risk:** Outcome measures were objective and standardized. | **Low Risk:** All pre‑specified outcomes were reported. | **High Risk:** High dropout rate and use of per‑protocol analysis result in significant bias in the overall findings. |
| **Horton et al. 2021** | **Some Concerns:** Randomization was performed, but deviations in analysis (completer analysis) increase bias. | **High Risk:** Dropout rate (35-41%) led to missing data and reduced comparability between groups. | **High Risk:** High dropout rate and differential attrition (especially in the control group) likely bias the results. | **Low Risk:** Objective outcome measurements were used, though the high attrition affects conclusions. | **Low Risk:** All outcomes were reported according to protocol. | **High Risk:** High dropout rate and missing data create significant bias, leading to high overall risk. |
| **Maltais et al., 2008** | **Low Risk:** Randomization and allocation concealment were appropriate. | **Low Risk:** No deviations from intended interventions. | **Low Risk:** Missing data was balanced across groups (14%). | **Low Risk:** Outcome measures were objective and standardized. | **Low Risk:** All pre‑specified outcomes were reported. | **Low Risk:** The study exhibited low risk in all domains. |
| **Güell et al., 2008** | **Low Risk:** Randomization was done, and baseline characteristics were balanced. | **High Risk:** The study excluded dropouts, leading to potential bias. | **High Risk:** High dropout rate (family group 17.9%, hospital group 3.4%) creates bias. | **Low Risk:** Objective outcome measures were used. | **Some Concerns:** High dropout rate and selective reporting could impact the reliability of results. | **High Risk:** The complete‑case analysis and differential dropout rates increase the overall risk of bias. |
| **Lahham et al., 2019** | **Low Risk:** Randomization and allocation concealment were well‑handled. | **High Risk:** 34% of participants were excluded, which could lead to bias. | **High Risk:** Non‑random missingness (MNAR) due to exclusion of non‑compliant participants likely biases the results. | **Low Risk:** Objective outcome measurements were used. | **Some Concerns:** The exclusion of participants and missing data could lead to selective reporting. | **High Risk:** Non‑random missingness and exclusion of participants undermine the overall findings. |
| **Horton et al. 2018** | **Low Risk:** Randomization and allocation concealment were properly executed. | **High Risk:** A high dropout rate (35-41%) introduced bias, especially with non-random missing data. | **High Risk:** Missing data and the use of a complete‑case analysis likely introduced bias. | **Low Risk:** Objective and standardized outcome measures. | **Low Risk:** All outcomes were reported as per protocol. | **High Risk:** High dropout rates and missing data introduce significant bias. |
| **Vasilopoulou et al., 2017** | **Some Concerns:** Randomization was performed correctly, and baseline characteristics were balanced. | **Low Risk:** No significant deviations from intended interventions. | **Low Risk:** Missing data was minimal, and no bias was detected. | **Some Concerns:** Outcome measurement was objective. | **Low Risk:** All outcomes were pre‑specified and reported transparently. | **Some Concerns:** Low bias across all domains, leading to low overall risk of bias. |
| **Holland et al., 2017** | **Low Risk:** Randomization and allocation concealment were properly performed. | **Low Risk:** No deviations from intended interventions. | **Low Risk:** Missing data was minimal (10%), and all outcomes were assessed. | **Low Risk:** Outcome measures were objective and standardized. | **Low Risk:** All pre‑specified outcomes were reported. | **Low Risk:** The study exhibited low risk in all domains. |
| **Mendes de Oliveira et al., 2010** | **Some Concerns:** Randomization was done, but 50% of the outpatient group was excluded, undermining comparability. | **High Risk:** The study excluded a disproportionate number of participants from the outpatient group (50%), leading to potential bias. | **High Risk:** Missing data and exclusion of participants introduce bias, especially in the outpatient group. | **Some Concerns:** Objective measurements were used, but differential dropout could lead to bias. | **Some Concerns:** Selective reporting is likely due to the high dropout rate. | **High Risk:** The exclusion of a large portion of the outpatient group and high dropout rates lead to significant bias. |
| **Sacristán-Galisteo et al., 2025** | **Low Risk:** Randomization and allocation concealment were properly handled. | **Low Risk:** No significant deviations from intended interventions. | **High Risk:** The dropout rate in the experimental group (25%) was higher than in the control group (12.5%), introducing bias. | **Low Risk:** Objective and standardized outcome measures were used. | **Low Risk:** All pre‑specified outcomes were reported. | **High Risk:** Differential dropout between groups introduces bias, especially for long-term follow‑up. |
