# Supplementary material for: Comparing Pulmonary Telerehabilitation and Center-Based Pulmonary Rehabilitation for Effectiveness and Adherence in Chronic Obstructive Pulmonary Disease: Systematic Review and Meta-Analysis of Randomized Controlled Trials
Source: J Med Internet Res. 2026 Apr 17;28:e80500. doi: 10.2196/80500 (PMC13089800; doi:10.2196/80500)
Supplement: Multimedia Appendix 7 [file jmir-v28-e80500-s007.docx]

| ****Multimedia Appendix 7.** Exploratory Subgroup Analyses of Clinical Outcomes** | | | | | | |
| --- | --- | --- | --- | --- | --- | --- |
| **Outcome — Subgroup** | **No. of studies**  **(k)** | **Sample size**  **(n)** | **Pooled MD—RR**  **(95% CI)** | ***P* value** | **Heterogeneity**  **(*I^2^*, %)** | ***P* subgroup​** |
| ***6MWD (End of intervention)*** | | | | | | |
| **delivery models** |  |  |  |  |  | .68 |
| digitally supported Tele-PR | 4 | 382 | -3.65 (-12.76 to 5.46) | .88 | 0.0 |  |
| low-technology HBPR | 5 | 568 | -7.11 (-28.80 to 14.59) | .03 | 61.2 |  |
| **supervision Intensity** |  |  |  |  |  | .92 |
| Asynchronous / Synchronous | 5 | 593 | -2.86 (−24.73 to 19.01) | .04 | 60.6 |  |
| Real-time Video / Synchronous Supervision | 2 | 250 | -4.55 (−31.39 to 22.30) | .74 | 0.0 |  |
| Minimal / Unsupervised | 1 | 51 | -11.00 (−49.68 to 27.68) | — | — |  |
| **supervision modalities** |  |  |  |  |  | .10 |
| Web/APP | 2 | 122 | -0.13 (−123.26 to 123.00) | .49 | 0.0 |  |
| Video | 2 | 250 | -4.55 (-31.39 to 22.30) | .74 | 0.0 |  |
| Other | 3 | 432 | 2.92 (-31.71 to 37.54) | .20 | 37.8 |  |
| Telephone | 1 | 80 | -24.30 (-41.18 to -7.42) | — | — |  |
| ***6MWD (long-term follow-up (≥6 months))*** | | | | | | |
| **delivery models** |  |  |  |  |  | .30 |
| digitally supported Tele-PR | 5 | 453 | 10.11 (-17.61 to 37.82) | .25 | 25.6 |  |
| low-technology HBPR | 4 | 495 | -3.58 (-31.84 to 24.68) | .08 | 60.0 |  |
| **supervision Intensity** |  |  |  |  |  | .33 |
| Asynchronous / Synchronous | 5 | 655 | −0.10 (−25.49 to 25.28) | .05 | 55.6 |  |
| Real-time Video / Synchronous Supervision | 2 | 242 | 14.84 (−51.32 to 80.99) | .57 | 0.0 |  |
| Minimal / Unsupervised | 1 | 23 | −1.00 (−36.65 to 34.65) | — | — |  |
| **supervision modalities** |  |  |  |  |  | .10 |
| Web/APP | 2 | 120 | 19.25 (-468.08 to 506.59) | .05 | 73.0 |  |
| Video | 2 | 242 | 14.84 (-51.32 to 80.99) | .57 | 0.0 |  |
| Other | 3 | 415 | 5.91 (-5.16 to 16.98) | .86 | 0.0 |  |
| Telephone | 2 | 171 | -15.72 (-195.65 to 164.22) | .12 | 57.9 |  |
| **Daily Steps (End of intervention)** | | | | | | |
| **delivery models** |  |  |  |  |  | .07 |
| digitally supported Tele-PR | 3 | 144 | 2.31 (-4.25 to 8.91) | .63 | 0.0 |  |
| low-technology HBPR | 2 | 109 | 10.43 (-43.43 to 64.30) | .28 | 15.0 |  |
| ***Daily Steps (long-term follow-up (≥6 months))*** | | | | | | |
| **delivery models** |  |  |  |  |  | .44 |
| digitally supported Tele-PR | 1 | 55 | -3.02 (-11.03 to 4.99) | — | — |  |
| low-technology HBPR | 2 | 87 | 0.22 (-11.04 to 11.48) | .78 | 0.0 |  |
| ***COPD Assessment Test (End of intervention)*** | | | | | | |
| **delivery models** |  |  |  |  |  | ＜.001 |
| digitally supported Tele-PR | 2 | 218 | -1.71 (-6.66 to 3.24) | .65 | 0.0 |  |
| low-technology HBPR | 1 | 80 | 4.20 (2.05 to 6.35) | — | — |  |
| ***COPD Assessment Test (long-term follow-up (≥6 months))*** | | | | | | |
| **delivery models** |  |  |  |  |  | ＜.001 |
| digitally supported Tele-PR | 3 | 294 | -0.10 (-1.77 to 1.57) | .76 | 0.0 |  |
| low-technology HBPR | 1 | 80 | 4.60 (2.32 to 6.88) | — | — |  |
| ***Chronic Respiratory Questionnaire–Dyspnea (End of intervention)*** | | | | | | |
| **delivery models** |  |  |  |  |  | .25 |
| digitally supported Tele-PR | 2 | 197 | -0.14 (-1.96 to 1.69) | 0.63 | 0.0 |  |
| low-technology HBPR | 4 | 594 | 0.21 (-0.64 to 1.06) | 0.13 | 47.0 |  |
| ***Chronic Respiratory Questionnaire–Dyspnea (long-term follow-up (≥6 months))*** | | | | | | |
| **delivery models** |  |  |  |  |  | .41 |
| digitally supported Tele-PR | 1 | 135 | -1.00 (-4.09 to 2.09) | — | — |  |
| low-technology HBPR | 4 | 551 | 0.18 (-0.25 to 0.60) | .35 | 8.1 |  |
| ***Dropout Rate (End of intervention)*** | | | | | | |
| **delivery models** |  |  |  |  |  | .25 |
| Tele-PR | 4 | 432 | 0.87 (0.31 to 2.46) | .002 | 80.0 |  |
| low-technology HBPR | 6 | 972 | 0.53 (0.26 to 1.08) | .002 | 73.4 |  |
| **supervision modalities** |  |  |  |  |  | .10 |
| Web / APP | 2 | 156 | 1.39 (0.01 to 170.89) | .09 | 66.0 |  |
| Video | 2 | 276 | 0.54 (0.02 to 15.42) | .27 | 18.7 |  |
| Other | 4 | 793 | 0.46 (0.15 to 1.38) | ＜.001 | 83.0 |  |
| Telephone | 2 | 179 | 0.89 (0.01 to 104.78) | .24 | 0.0 |  |

|  |
| --- |

|  |
| --- |

|  |
| --- |


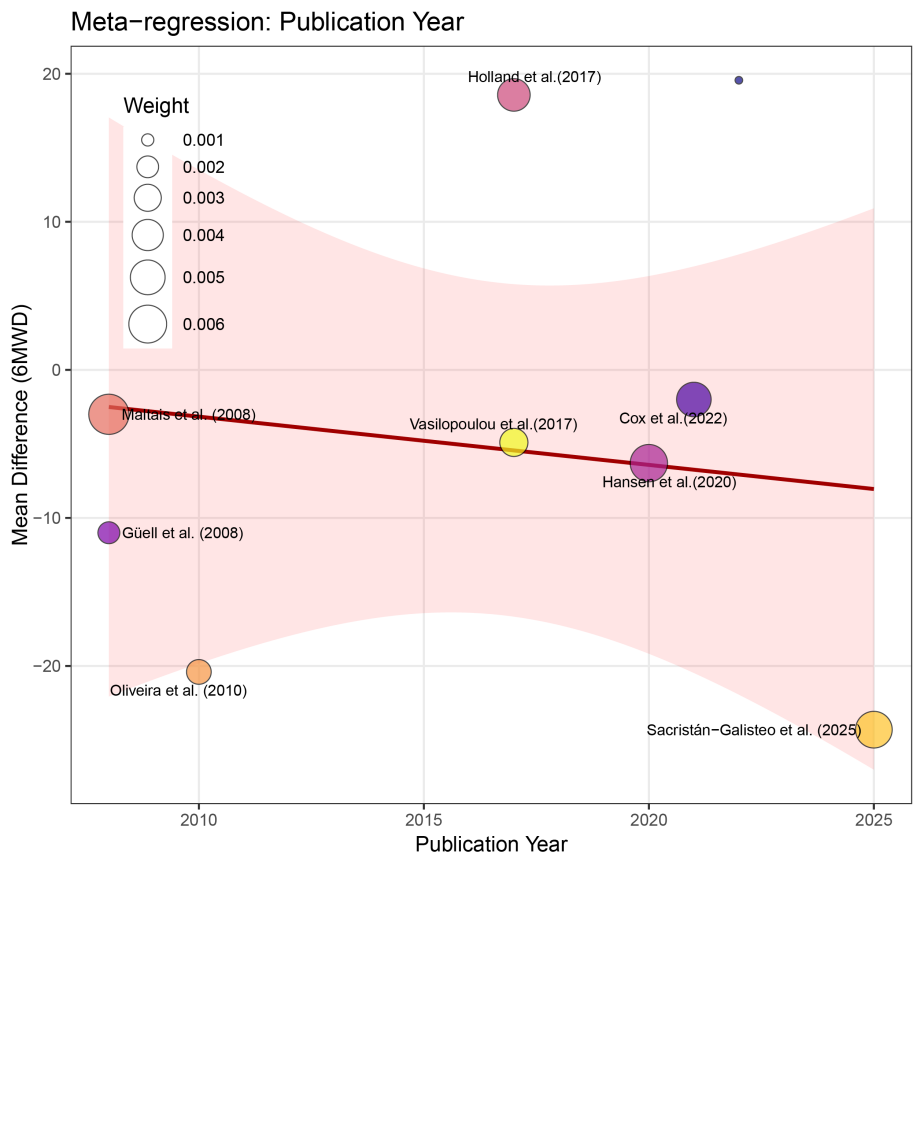


**Figure S7.1** Bubble plot of meta-regression for the association between Publication Year and mean difference in 6MWD at the end of intervention.


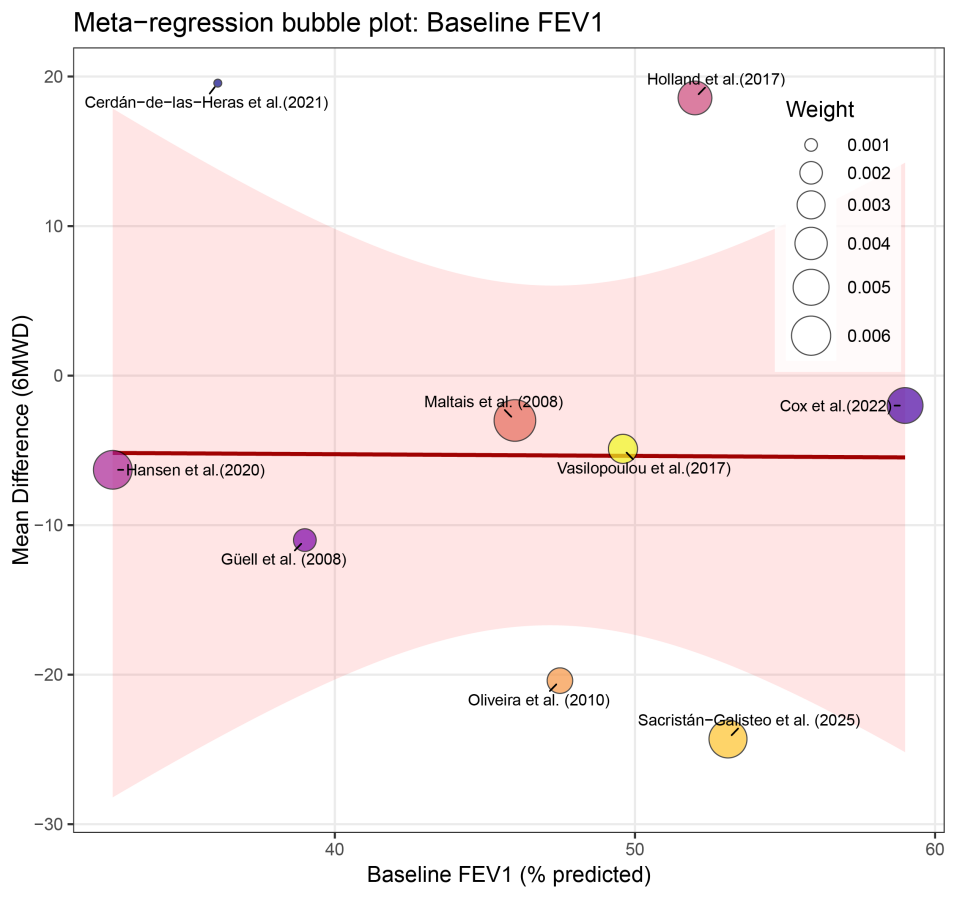


**Figure S7.2**. Bubble plot of meta-regression for the association between baseline FEV_1_ (% predicted) and mean difference in 6MWD at the end of intervention.


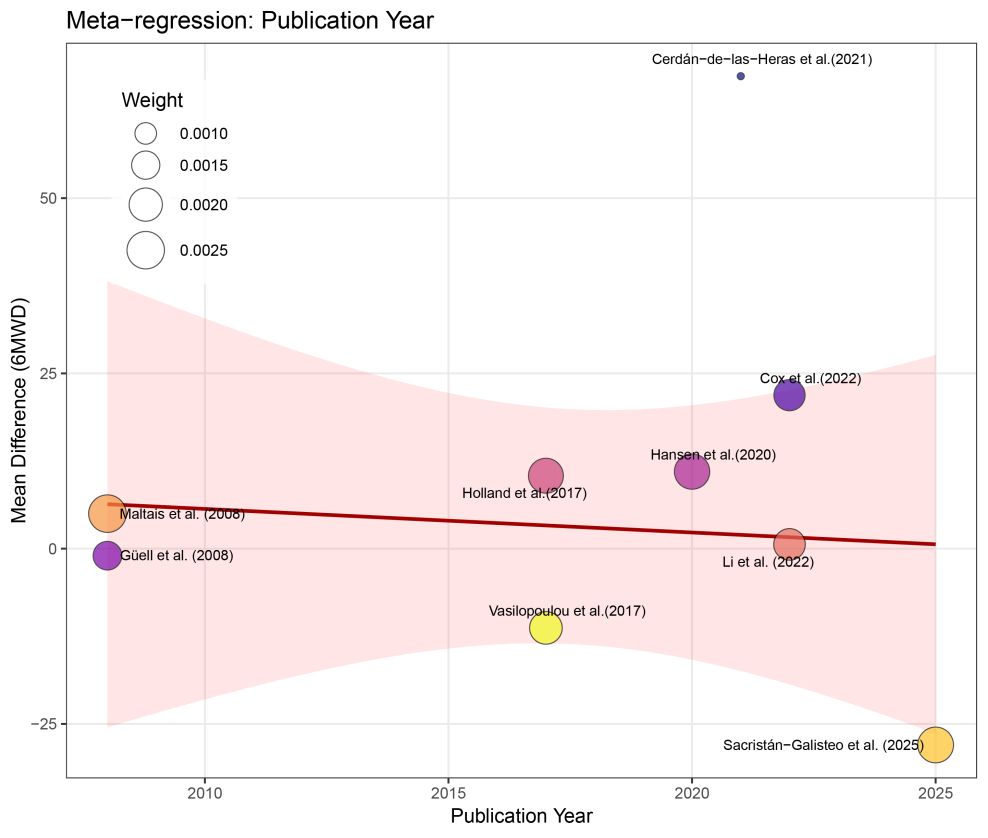


**Figure S7.3**. Bubble plot of meta-regression for the association between Publication Year and mean difference in 6MWD at long-term follow-up (≥6 months).


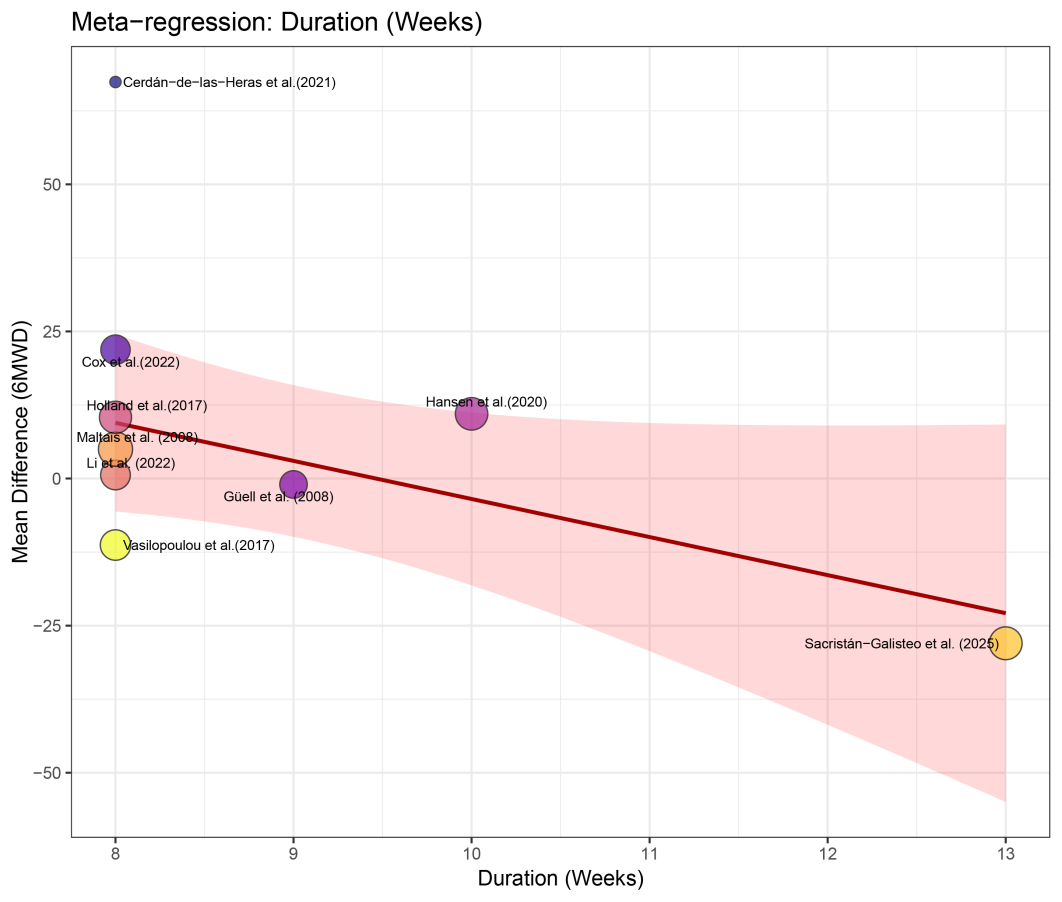


**Figure S7.4**. Bubble plot of meta-regression for the association between Duration weeks and mean difference in 6MWD at long-term follow-up (≥6 months).


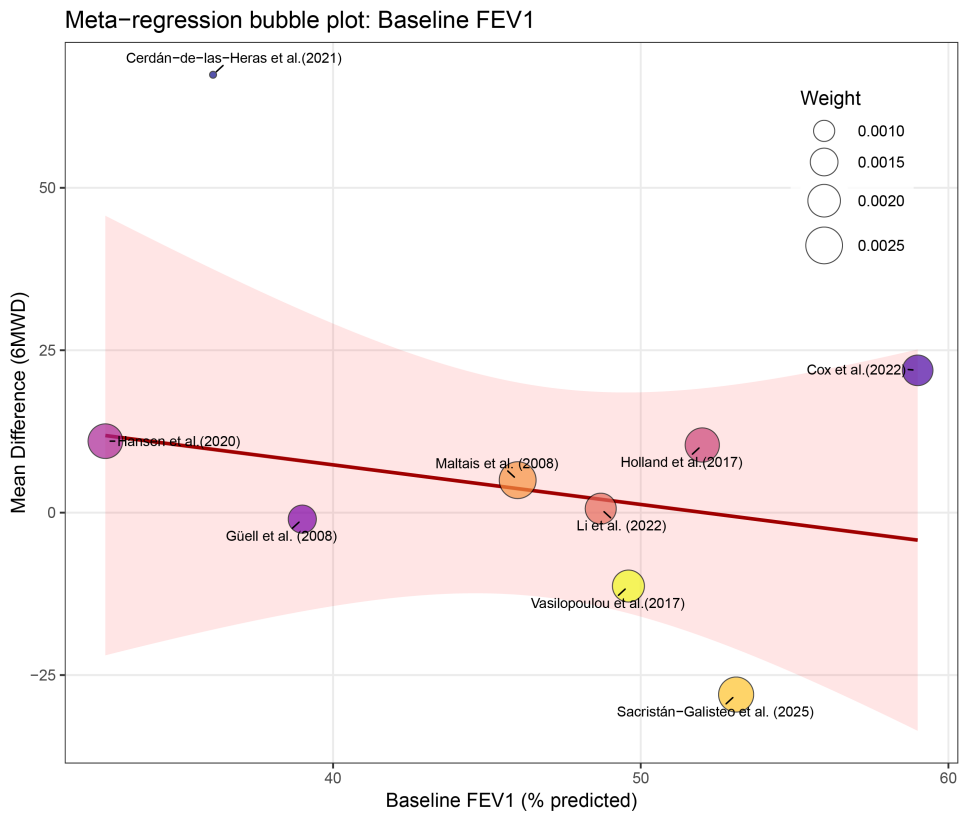


**Figure S7.5**. Bubble plot of meta-regression for the association between baseline FEV_1_ (% predicted) and mean difference in 6MWD at long-term follow-up (≥6 months).


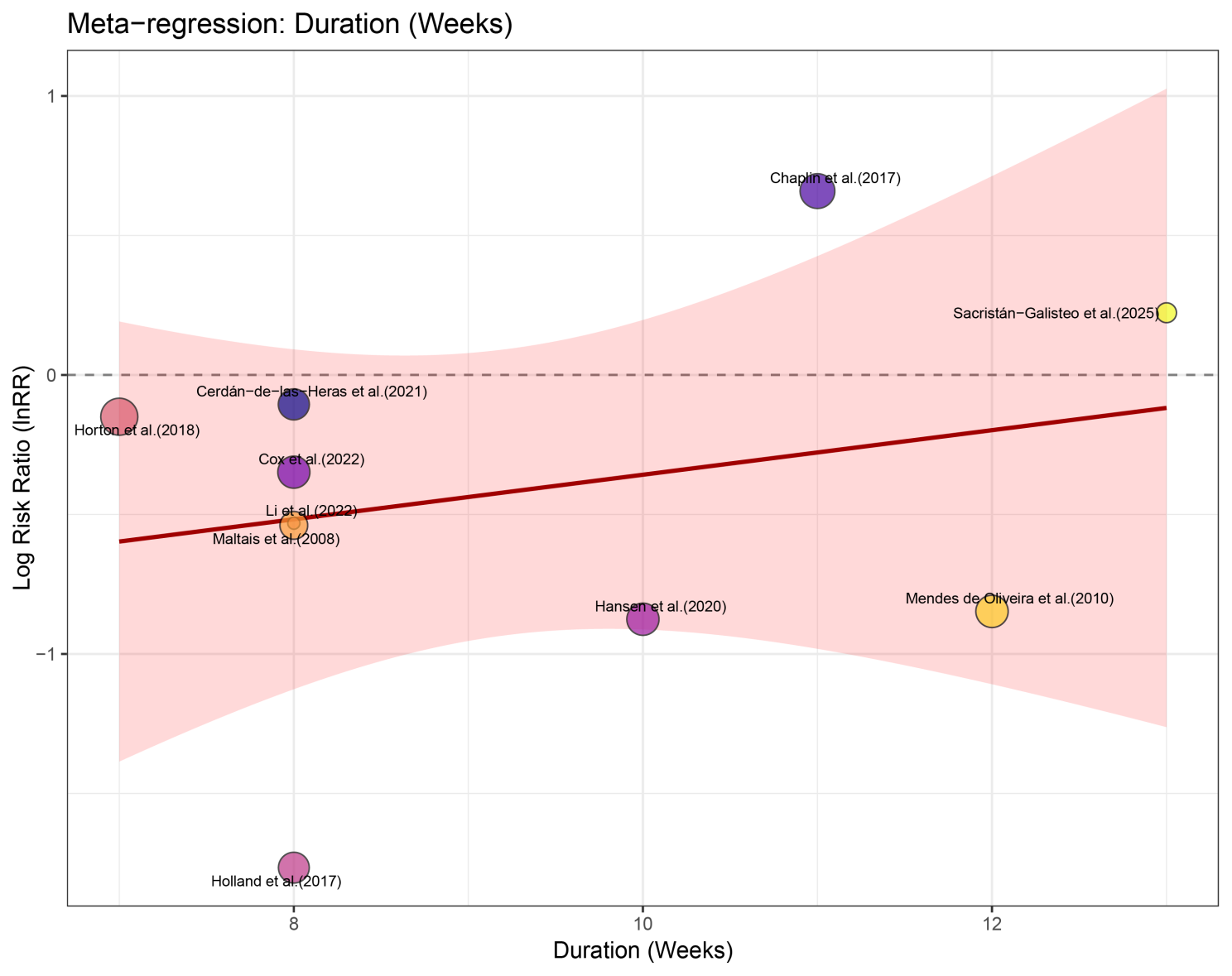


**Figure S6.** Bubble plot of meta-regression for the association between Duration weeks and risk ratio in dropout rates at the end of intervention.


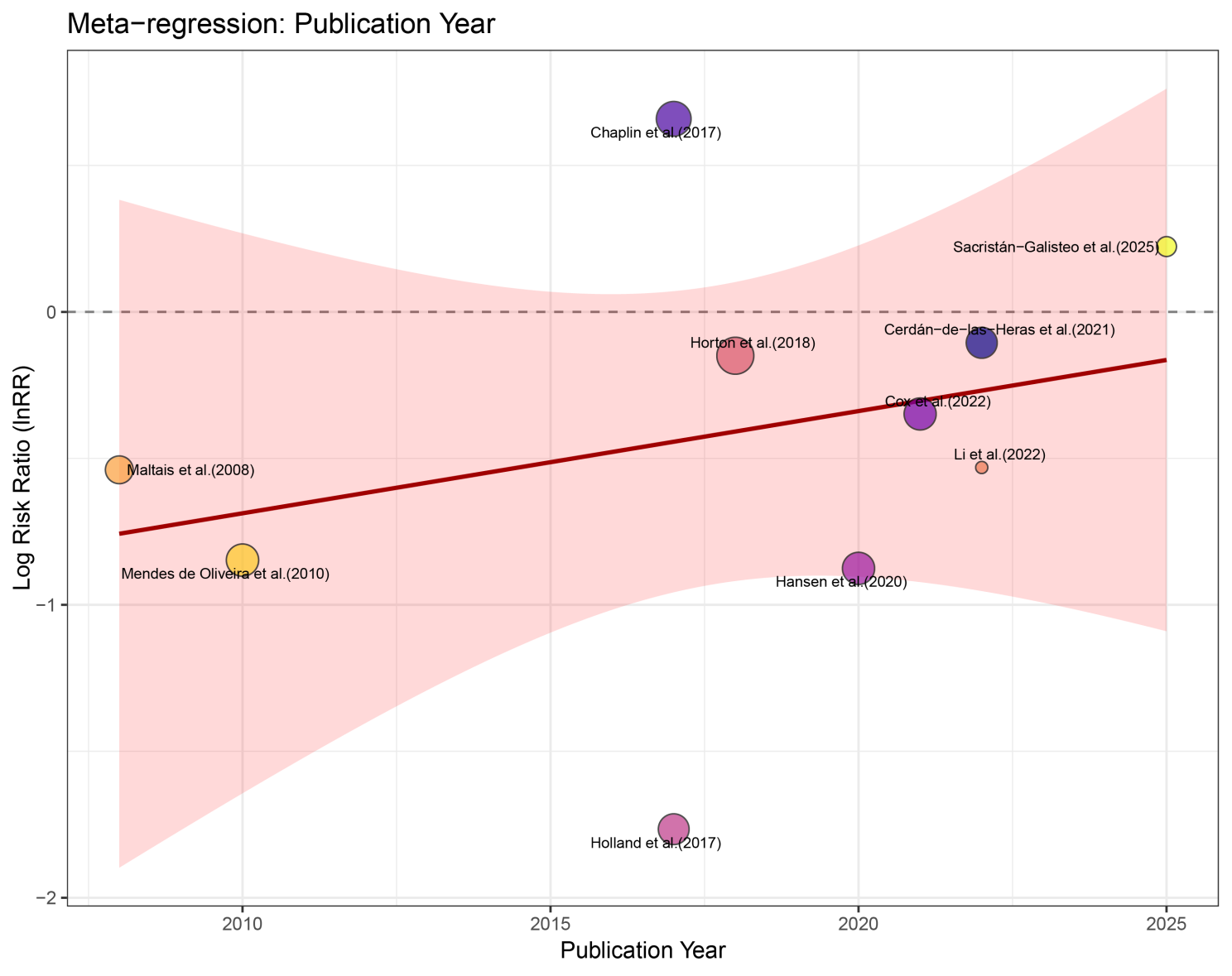


**Figure S7**. Bubble plot of meta-regression for the association between Publication Year and risk ratio in dropout rates at the end of intervention.
