# Supplementary material for: Comparing Pulmonary Telerehabilitation and Center-Based Pulmonary Rehabilitation for Effectiveness and Adherence in Chronic Obstructive Pulmonary Disease: Systematic Review and Meta-Analysis of Randomized Controlled Trials
Source: J Med Internet Res. 2026 Apr 17;28:e80500. doi: 10.2196/80500 (PMC13089800; doi:10.2196/80500)
Supplement: Multimedia Appendix 8 [file jmir-v28-e80500-s008.docx]

Table 8.1 Leave-One-Out Sensitivity Analysis for 6MWD End-Intervention

| Omitted Study | MD (m) | 95% CI | *P* value | *I²* (%) | τ² |
| --- | --- | --- | --- | --- | --- |
| None (overall) | −5.37 | −15.68 to 4.95 | .26 | 28.2 | 103.97 |
| Cerdán-de-las-Heras et al. (2021) | −5.98 | −16.84 to 4.87 | .23 | 33.4 | 92.38 |
| Hansen et al. (2020) | −5.15 | −17.53 to 7.24 | .36 | 37.1 | 124.45 |
| Cox et al. (2022) | −5.85 | −18.02 to 6.31 | .29 | 36.2 | 123.54 |
| Vasilopoulou et al. (2017) | −5.37 | −17.30 to 6.56 | .32 | 37.1 | 124.76 |
| Maltais et al. (2008) | −5.85 | −18.37 to 6.67 | .31 | 35.8 | 124.10 |
| Güell et al. (2008) | −4.98 | −16.62 to 6.65 | .35 | 36.7 | 121.26 |
| ****Holland et al. (2017)**** | ****−8.63**** | ****−17.03 to −0.24**** | ****.045**** | ****0.0**** | ****54.54**** |
| Oliveira et al. (2010) | −4.26 | −15.34 to 6.82 | .39 | 32.5 | 101.29 |
| Sacristán-Galisteo et al. (2025) | −1.88 | −11.04 to 7.28 | .64 | 0.0 | 67.27 |

Table 8.2 Leave-One-Out Sensitivity Analysis for 6MWD Long-Term Outcomes

(≥6 Months Follow-up)

| Omitted Study | MD (m) | 95% CI | *P* value | *I²* (%) | τ² |
| --- | --- | --- | --- | --- | --- |
| None (overall) | 2.97 | −12.22 to 18.17 | .66 | 44.5 | 319.32 |
| Cerdán-de-las-Heras et al. (2021) | 0.60 | −12.51 to 13.71 | .92 | 38.6 | 134.29 |
| Li et al. (2022) | 3.51 | −30.38 to 31.58 | .66 | 51.4 | 380.23 |
| Hansen et al. (2020) | 2.00 | −14.38 to 21.41 | .80 | 48.3 | 376.84 |
| Cox et al. (2022) | 0.81 | −46.92 to 53.95 | .91 | 44.0 | 339.99 |
| Vasilopoulou et al. (2017) | 4.92 | −15.88 to 19.89 | .52 | 48.3 | 341.89 |
| Maltais et al. (2008) | 2.90 | −12.19 to 22.03 | .72 | 50.6 | 383.16 |
| Güell et al. (2008) | 3.62 | −15.38 to 21.18 | .64 | 51.4 | 378.02 |
| Holland et al. (2017) | 2.12 | −14.08 to 21.32 | .79 | 48.9 | 378.08 |
| **Sacristán-Galisteo et al. (2025)** | **7.48** | **−5.16 to 20.12** | **.20** | **0.0** | **201.08** |

Table 8.3 Leave-One-Out Sensitivity Analysis for CAT End-Intervention

| Omitted study | MD | 95% CI | *P* value | *I²* (%) | τ² |
| --- | --- | --- | --- | --- | --- |
| None (overall) | 0.24 | −8.44 to 8.92 | .92 | 89.1 | 10.42 |
| Hansen et al. (2020) | 1.06 | −16.07 to 16.55 | .80 | 92.5 | 17.81 |
| Vasilopoulou et al. (2017) | 1.40 | −39.60 to 41.71 | .71 | 92.4 | 13.62 |
| **Sacristán-Galisteo et al. (2025)** | **−1.71** | **−6.66 to 3.24** | **.14** | **0** | **0.03** |

Table 8.4 Leave-One-Out Sensitivity Analysis for mMRC End-Intervention

| Omitted study | MD | 95% CI | *P* value | *I²* (%) | τ² |
| --- | --- | --- | --- | --- | --- |
| None (overall) | 0.09 | −0.78 to 0.95 | .71 | 74.8 | 0.09 |
| Cox et al. (2022) | 0.18 | −1.46 to 1.64 | .66 | 84.3 | 0.15 |
| **Vasilopoulou et al. (2017)** | **−0.11** | **−3.75 to 4.12** | **.06** | **0** | **<0.001** |
| Holland et al. (2017) | 0.19 | −3.62 to 4.00 | .63 | 83.3 | 0.13 |

Table 8.5 Leave-One-Out Sensitivity Analysis for CRQ-D End-Intervention

| Omitted study | MD | 95% CI | *P* value | *I²* (%) | τ² |
| --- | --- | --- | --- | --- | --- |
| None (overall) | 0.10 | −0.39 to 0.60 | .62 | 24.8 | 0.22 |
| Chaplin et al. (2017) | 0.16 | −0.53 to 0.86 | .55 | 34.5 | 0.28 |
| Cox et al. (2022) | 0.13 | −0.43 to 0.70 | .55 | 35.8 | 0.21 |
| Maltais et al. (2008) | 0.13 | −0.61 to 0.87 | .64 | 35.9 | 0.30 |
| Güell et al. (2008) | 0.18 | −0.47 to 0.84 | .48 | 28.4 | 0.26 |
| **Horton et al. (2018)** | **0.02** | **−0.66 to 0.70** | **.94** | **0** | **0.28** |
| Holland et al. (2017) | 0.06 | −0.27 to 0.39 | .63 | 7.3 | 0.05 |

Table 8.6 Leave-One-Out Sensitivity Analysis for Drop rate End-Intervention

| Omitted study | RR | 95% CI | P value | *I²* (%) | τ² |
| --- | --- | --- | --- | --- | --- |
| None (overall) | 0.66 | 0.40 to 1.07 | 0.08 | 76.4 | 0.34 |
| Chaplin et al. (2017) | 0.57 | 0.37 to 0.88 | 0.02 | 62.6 | 0.21 |
| Cerdán-de-las-Heras et al. (2021) | 0.63 | 0.37 to 1.10 | 0.09 | 78.8 | 0.37 |
| Hansen et al. (2020) | 0.69 | 0.40 to 1.19 | 0.16 | 76.9 | 0.36 |
| Li et al. (2022) | 0.66 | 0.38 to 1.14 | 0.12 | 78.9 | 0.39 |
| Cox et al. (2022) | 0.65 | 0.37 to 1.14 | 0.11 | 79.0 | 0.39 |
| Maltais et al. (2008) | 0.66 | 0.38 to 1.15 | 0.13 | 78.8 | 0.38 |
| Horton et al. (2018) | 0.63 | 0.36 to 1.10 | 0.09 | 78.3 | 0.37 |
| Holland et al. (2017) | 0.78 | 0.52 to 1.16 | 0.18 | 62.6 | 0.17 |
| Mendes de Oliveira et al. (2010) | 0.69 | 0.40 to 1.19 | 0.16 | 77.0 | 0.36 |
| Sacristán-Galisteo et al. (2025) | 0.63 | 0.37 to 1.06 | 0.08 | 78.6 | 0.35 |

Table 8.7 Leave-One-Out Sensitivity Analysis for Daily Steps End-Intervention

| Omitted study | MD  (per 100 steps/day) | 95% CI | P value | *I²* (%) | τ² |
| --- | --- | --- | --- | --- | --- |
| None (overall) | 4.97 | −1.84 to 11.78 | .11 | 23.8 | 18.05 |
| erdán-de-las-Heras et al. (2021) | 6.39 | to 1.56–14.35 | 0.08 | 14.5 | 12.95 |
| Chaplin et al. (2022) | 5.04 | to 5.04–15.11 | 0.21 | 41.9 | 26.63 |
| Hansen et al. (2020) | 5.97 | to 4.37–16.32 | 0.16 | 38.1 | 23.54 |
| Holland et al. (2017) | 4.74 | to 5.31–14.80 | 0.23 | 39 | 26.04 |
| **Horton et al. (2021)** | **3.11** | **−1.71 to 7.93** | **0.13** | **00.0** | **3.23** |
